# Comparative analysis of *Streptococcus suis* genomes identifies novel candidate virulence-associated genes in North American isolates

**DOI:** 10.1186/s13567-022-01039-8

**Published:** 2022-03-18

**Authors:** April A. Estrada, Marcelo Gottschalk, Connie J. Gebhart, Douglas G. Marthaler

**Affiliations:** 1 Department of Veterinary and Biomedical Sciences, College of Veterinary Medicine, University of Minnesota, Saint Paul, MN USA; 2 Faculty of Veterinary Medicine, University of Montreal, Saint-Hyacinthe, QC Canada; 3 Independent Researcher, Kansas State University, Kansas City, KS USA

**Keywords:** *Streptococcus suis*, virulence-associated genes (VAGs), pathotype, comparative genomics

## Abstract

*Streptococcus suis* is a significant economic and welfare concern in the swine industry. Pan-genome analysis provides an in-silico approach for the discovery of genes involved in pathogenesis in bacterial pathogens. In this study, we performed pan-genome analysis of 208 *S. suis* isolates classified into the pathogenic, possibly opportunistic, and commensal pathotypes to identify novel candidate virulence-associated genes (VAGs) of *S. suis*. Using chi-square tests and LASSO regression models, three accessory pan-genes corresponding to *S. suis* strain P1/7 markers *SSU_RS09525*, *SSU_RS09155*, and *SSU_RS03100* (>95% identity) were identified as having a significant association with the pathogenic pathotype. The proposed novel *SSU_RS09525* + */SSU_RS09155* + */SSU_RS03100* + genotype identified 96% of the pathogenic pathotype strains, suggesting a novel genotyping scheme for predicting the pathogenicity of *S. suis* isolates in North America. In addition, mobile genetic elements carrying antimicrobial resistance genes (ARGs) and VAGs were identified but did not appear to play a major role in the spread of ARGs and VAGs.

**Supplementary Information:**

The online version contains supplementary material available at 10.1186/s13567-022-01039-8.

## Introduction

*Streptococcus suis* is a significant economic and welfare concern in the swine industry as pathogenic strains cause a range of clinical signs including meningitis, arthritis, endocarditis, and septicemia [1]. Commensal strains, however, naturally colonize the upper respiratory tract of pigs without causing clinical disease [2]. Characterization techniques capable of identifying strains of clinical significance are important for the control of *S. suis* disease. Virulence-associated factors contributing to the virulence of *S. suis* strains (mainly for serotype 2) have been described but are not consistently present in clinical isolates [3, 4].

Increasing availability of next generation sequencing technologies and generation of large amounts of data led to the development of numerous programs and software tools for bacterial typing [5, 6]. Pan-genome analysis characterizes the diversity within a bacterial species as it describes core traits shared by all strains and unique/accessory traits shared only by some strains [7]. *S. suis* possesses an open pan-genome in which the number of unique or accessory genes increases as more genomes are sequenced [8]. The open pan-genome contributes to high species diversity and is typically indicative of high rates of horizontal gene transfer by mobile genetic elements (MGEs) [7, 9]. Functional analyses of the *S. suis* pan-genome revealed differences in the functional annotation of the core genome compared to the accessory genome [8, 10], supporting the distinct roles of these two components in explaining genomic diversity. Comparative genomics can also be used as a subtyping tool for *S. suis*. Serotype 1, 2, 7, and 9 strains were differentiated by comparative genome hybridization or bayesian analysis of population structure into multiple genomic groups, some of which correlated to virulence and traditional molecular subtypes [11, 12].

The ability to identify virulence determinants within the accessory genome has been demonstrated for various bacterial pathogens such as *Escherichia coli*, *Pseudomonas aeruginosa*, and *Streptococcus* spp. [13–15]. Known and putative VAGs were over-represented in systemic isolates of *S. suis* compared to respiratory and non-clinical isolates [16]. A genome-wide association study was utilized to identify three novel genetic markers for differentiating *S. suis* isolates into invasive disease-associated and non-disease-associated groups [17]. That pathotyping tool, which consisted of a multiplex-PCR assay, demonstrated performance metrics (e.g., sensitivity, specificity) similar to serotyping, multilocus sequence typing (MLST), VAG profiling, and minimum core genome sequence typing. Two recent publications on the comparative analysis of virulent and avirulent strains identified two different sets of novel virulence-associated genes, supporting previous claims of an abundance of uncharacterized virulence determinants in the accessory genome of pathogenic strains of *S. suis* [10, 18].

Mobile genetic elements facilitate intracellular and intercellular movement of genes and contribute to the evolution of pathogenic bacteria such as *S. suis* [19–21]*.* Proteins encoded by MGEs have various functions that can be described as core traits required for replication and advantageous/adaptive traits [e.g., antimicrobial resistance genes (ARGs), virulence-associated genes (VAGs)] [19, 22]. Previous studies demonstrated the transfer of conjugative ICE*Ssu*32457 to *S. agalactiae*, *S. pneumoniae*, and *S. pyogenes*, indicating the dissemination of resistance genes among closely related species [23, 24]. An 89 kb PAI was first identified in the highly virulent and zoonotic Chinese *S. suis* strain 05ZYH33, and it contains both resistance genes and putative VAGs [25, 26]. A high degree of phenotypic antimicrobial resistance was associated with the presence of plasmids in *S. suis* [27]. However, only a few resistance genes, such as the multiresistance gene *cfr* and the chloramphenicol resistance gene *cmr*, have been identified on *S. suis* plasmids [28, 29]. There is evidence that *S. suis* serves as an MGE reservoir for other streptococci, and there are many poorly characterized MGEs [21, 30].

Comparative genomics provides a powerful tool for the characterization and subtyping of *S. suis*. However, a contemporary comparative pan-genomic study targeting U.S. *S. suis* strains is unavailable. We performed comparative genome analysis on a set of 208 *S. suis* isolates from North America (mainly the United States) to identify accessory genes corresponding to the pathogenic pathotype that may thus serve as novel candidate virulence-associated genes of *S. suis*. The identification of candidate VAGs may elucidate a novel VAG genotyping scheme for predicting the pathogenicity of *S. suis* isolates in North America. Further, we performed a preliminary analysis of the diversity of MGEs in *S. suis* isolates and their role in the dissemination of ARGs and VAGs among *S. suis* pathotypes.

## Materials and methods

### Sample selection and genome assembly

The study utilized all 208 *S. suis* isolates (referred to as the training set) recovered from pigs in North America (mainly the United States: U.S., *n* = 203; Canada, *n* = 4; Mexico, *n* = 1) and previously described by Estrada et al. [31]. These isolates were previously classified into pathotypes (pathogenic, possibly opportunistic, and commensal) and characterized by serotyping, MLST, and VAG profiling. Pathogenic isolates (*n* = 139) were obtained from systemic tissues of diseased pigs in which *S. suis* was reported in the diagnostic report as the primary cause of disease. Possibly opportunistic isolates (*n* = 47) were predominantly obtained from lung tissues of pigs without signs of neurological or systemic disease, and commensal isolates (*n* = 22) were obtained from laryngeal, tonsil, or nasal samples of healthy pigs. Genome assembly was performed on Illumina sequencing data of the 208 *S. suis* isolates. Genome assemblies (contigs) were generated using the SKESA *de-novo* assembler (v2.4.0) [32] with default kmer settings. QUAST (v4.5) [33] was used to evaluate the genome assemblies and generate summary statistics (e.g. genome length, GC content, N50) for contigs ≥ 500 bp. Genome contamination and completeness were evaluated utilizing the CheckM taxonomic-specific (species) workflow [34]. Only contigs ≥ 500 bp were kept for annotation by Prokka (v1.14.6) [35] to predict coding sequences. The pan-genome was annotated using Roary (v3.13.0) [36] using a 90% BLASTp identity cut-off to define clusters of genes and allowing paralog clustering. Gene clusters present in 99% (≥ 206/208) of genomes were classified as core genes. Two different Roary analyses were performed. The first analysis utilized all 208 genomes while the second analysis utilized the 161 genomes representing only the pathogenic and commensal pathotypes.

### Functional annotation

The Clusters of Orthologous Groups of proteins (COG) database (2014) [37] was utilized to predict protein functions. For each gene cluster identified by Roary, a representative protein sequence was selected, and BLASTp searches against the COG database were performed. COG functional classes from searches meeting the thresholds (BLASTp, coverage ≥ 70%, identity ≥ 70%, and e-value ≤ 10–5) were plotted in R (v3.6.1) using Rstudio [38].

### Statistical analysis

Associations between *S. suis* accessory genes and pathotype were investigated in R as follows. The data were filtered by removing genes detected in less than 50% (≤ 69/139) of isolates within the pathogenic pathotype and detected in more than 50% (≥ 11/22) of isolates within the commensal pathotype. Remaining accessory genes were individually tested by chi-square using a 3 × 2 table comparing the three pathotypes and the status (presence/absence) of individual genes. Genes lacking a significant (*p*-value < 0.05) association with pathotype were removed from the analysis. The remaining genes were analyzed using the LASSO (Least Absolute Shrinkage and Selection Operator) shrinkage regression model.

### LASSO shrinkage regression model

**Figure 1:**
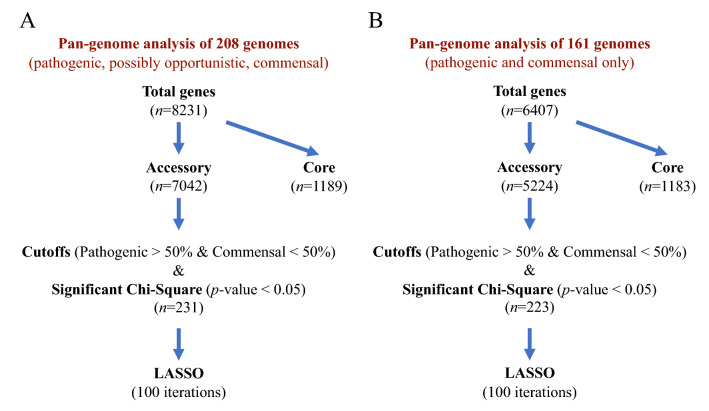
**Illustration of the pan-genome approach for identifying candidate virulence-associated genes (VAGs).** **A** The first data set analyzed consisted of all 208 isolates and **B** the second data set consisted of a subset of 161 isolates. Genes with a significant association (*p*-value < 0.05) with pathotype, and present in more than 50% (69/139) of isolates within the pathogenic pathotype and present in less than 50% (11/22) of isolates within the commensal pathotype, were analyzed using the LASSO shrinkage regression model. Genes identified in both LASSO analyses were selected as the ‘best’ predictors of pathotype.

A LASSO shrinkage regression model was used, as previously described by Estrada et al. [39], to determine the fewest number of accessory genes that may serve as predictors of pathogenicity; the pathogenic pathotype served as the indicator of pathogenicity. The LASSO analysis (100 iterations) was performed on each Roary data set, the first data set consisting of all 208 isolates, and the second consisting of a subset of 161 isolates (Figure 1). Genes identified in both LASSO analyses were selected as the 'best' predictors of pathogenicity.

### Identification of antimicrobial resistance genes

The presence of antimicrobial resistance genes (ARGs) was predicted in all 208 *S. suis* draft genomes (contigs) using the Comprehensive Antibiotic Resistance Database (CARD) (v3.1.0) and the CARD BLAST command-line program [40] (BLASTn, ≥ 90% identity and ≥ 60% coverage).

### Identification of mobile genetic elements

Plasmids and other genetic elements (insertion sequences, ICE, IME, composite transposons, etc.) were identified in the *S. suis* draft genomes using the command-line PlasmidFinder [41] and MobileElementFinder [42] programs (default parameters: ≥ 90% identity and ≥ 60% coverage). The PlasmidFinder database (07-13-2020), which is a curated database of plasmid replicons, was updated to include *S. suis* plasmid replicons (pNSUI060a: CP012912, pNSUI060b: CP012913, HN105 unnamed plasmid1: CP029399, pSRD478: CP017089, pISU2812: CP017093, pISU2514: CP030021, pISU2614: CP031378, pYSJ17: CP032065) available from NCBI. Plasmid draft sequences were extracted from the genomes by mapping the trimmed reads to the respective plasmid reference. Potentially novel plasmids were identified utilizing viralVerify [43] and the Pfam-A database (v35.0). Phages were identified and annotated utilizing PHASTER [44] and the location (chromosome, plasmid) of identified phages was investigated using the viralVerify data. The MobileElementFinder database (06-09-2020) contains genetic elements from several public databases including RefSeq, Tn registry and ICEberg. The presence of the *S. suis* strain 05ZYH33 89 K candidate pathogenicity island (PAI) was determined in-silico by screening for the CH1/CH2 (CP000407.1: 871,777–873,837 bp), CH3/CH4 (CP000407.1: 921,759–922,474 bp) and CH5/CH6 (CP000407.1: 961,264–962,239 bp) DNA sequences targeted by the PCR primers described by Schmid et al. [45]. Screening of these sequences was performed using the SRST2 (Short Read Sequence Typing for Bacterial Pathogens) program (≥ 90% coverage and ≥ 90% sequence identity) [46]. The CH3 and CH4 sequence is a 716 bp fragment unique to the strain 05ZYH33 89 K PAI, thus isolates lacking this segment were considered negative for the PAI. PAI draft sequences were extracted from the genomes by mapping the trimmed reads to the *S. suis* strain 05ZYH33 89 K PAI [47]. The plasmid, ICE, IME, composite transposon, and 89 K PAI draft sequences were annotated using Prokka. Associations between the presence of MGEs and pathotype were tested by chi-square in Rstudio.

### Identification of MGE-associated ARGs and VAGs

Comparative analysis to identify genetic elements carrying ARGs and putative VAGs was performed by BLAST searching MGE draft sequences against custom databases [BLASTn (dc-megablast), ≥ 80% identity and ≥ 60% coverage]. The CARD (v3.1.0) [40], CGE ResFinder (02-19-2021) [48], and ARG-ANNOT ARG (v6) [49] databases were combined into a custom ARG database. The VF (03-01-2021) [50], CGE VirulenceFinder (05-29-2020) [51], and *S. suis* VAG databases [39] were combined into a custom VAG database.

## Results

### Identification of core and accessory gene content

**Figure 2:**
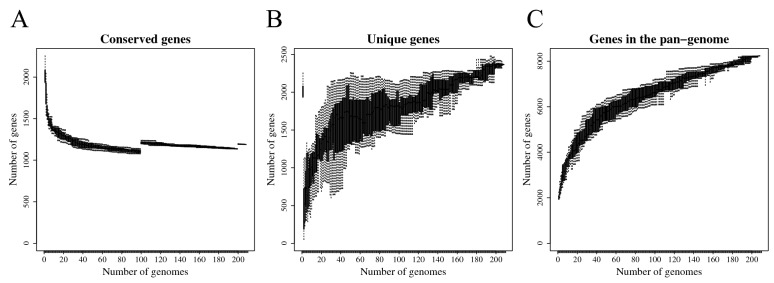
**Summary statistics for the pan-genome analysis of 208 *S. suis* isolates.** **A** Number of conserved genes, **B** unique genes, and **C** total number of genes plotted against the number of genomes included in the analysis.

In the current study, the pan-genome (core and accessory genes) of the training set of *S. suis* isolates was determined. The genome lengths ranged from 1.95 to 2.45 Mb with an average coverage of 208 × across the assembly, an average GC content of 41.2%, less than 5% genome contamination, and over 94.0% genome completeness (Additional file 1). The number of predicted protein coding sequences ranged from 1854 to 2399, with an average of 2078, and an average of 3 and 39 rRNAs and tRNAs, respectively. A total of 8231 gene clusters were identified (Additional file 2). Of these, 1189 gene clusters were classified as core genes and were present in all or nearly all genomes (≥ 206/208). A decrease in the number of conserved or core genes was observed as more genomes were added to the analysis (Figure 2A) while an increase in the number of unique genes was observed (Figure 2B). Furthermore, the total number of genes in the pan-genome continued to increase with each additional genome (Figure 2C), suggesting an open pan-genome for *S. suis* and indicating a potential to discover novel genes with the sequencing of more *S. suis* strains. These genome lengths, CG content, and number of predicted protein sequences are consistent with the *S. suis* reference strains already in the GenBank/EMBL/DDBJ database.

### Cluster of Orthologous Groups classification

**Figure 3:**
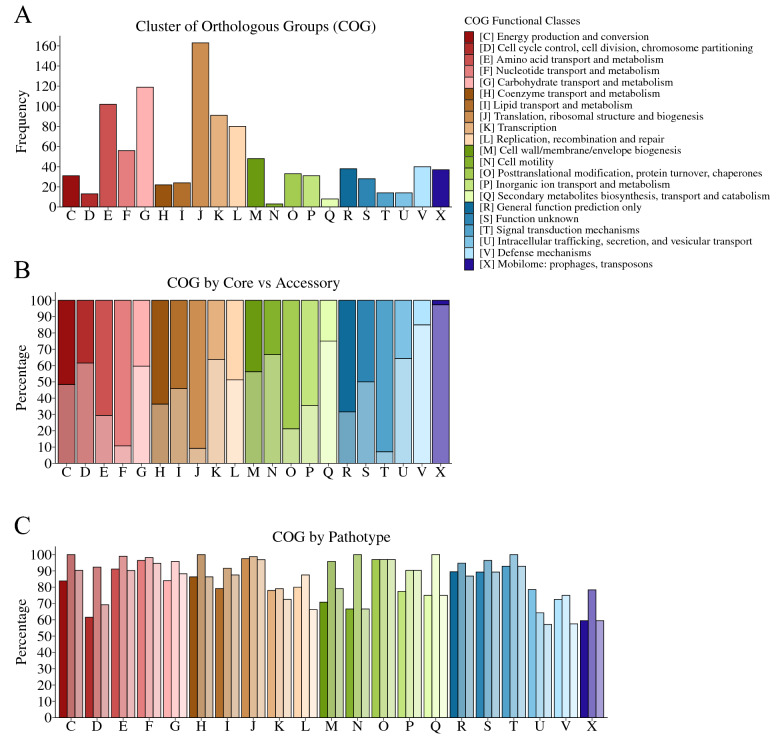
**Predicted Cluster of Orthologous Groups (COG) functional classes.** COG functional classes were determined for the 995 pan-gene clusters that met the criteria (psiblast, coverage ≥ 70%, percent identity ≥ 70%, and e-value ≤ 10–5). **A** The total frequencies of pan-gene clusters classified into each COG functional class (single-letter code) were plotted and color-coded. **B** The percentages of accessory and core pan-gene clusters classified into each COG functional class were plotted. Dark and light shading represents core and accessory pan-gene clusters, respectively. **C** The percentages of pan-gene clusters (from each COG class) present in each pathotype. Dark, medium, and light shading represents pan-gene clusters present in the pathogenic, possibly opportunistic, and commensal pathotypes, respectively.

The COG functional classes were predicted for 98.7% (8123/8231) of gene clusters identified in the pan-genome of *S. suis.* The comparison of COG functional classes between core and accessory genes was performed only on the 995 classifications that met the criteria (coverage ≥ 70%, percent identity ≥ 70%, and e-value ≤ 10–5) (Figure 3A, Additional file 3). Core genes were more likely to be classified into functional classes T (Signal transduction mechanisms), J (Translation, ribosomal structure and biogenesis), F (Nucleotide transport and metabolism), and O (Posttranslational modification, protein turnover, chaperones), in decreasing order. More accessory genes were classified into classes X (Mobilome: prophages, transposons) and V (Defense mechanisms), in decreasing order (Figure 3B). Major differences were lacking in the distribution of COG classes by pathotype (Figure 3C).

### Candidate VAGs

Statistical analyses were performed to test for associations between accessory genes and the three pathotypes. Of the 7,042-accessory pan-genes identified for the training set (3 pathotypes), 231 pan-genes met the criteria (*p*-value < 0.05, < 50% commensal, > 50% pathogenic, Figure 1A) to be further analyzed by the LASSO model. Further analysis was performed on the commensal and pathogenic pathotypes (*n* = 161) (Additional file 4), excluding the possibly opportunistic pathotype as it may contain true pathogenic or commensal strains, and 223 pan-genes were associated with the pathogenic pathotype and further analyzed by the LASSO model (Figure 1B). Four genes corresponding to *S. suis* strain P1/7 markers *SSU_RS09525*, *SSU_RS09155*, *SSU_RS03100*, and *SSU_RS01590* (>95% identity) were identified in both LASSO analyses (Table 1) and were further investigated as novel predictors of pathotype. The *SSU_RS09525* + */SSU_RS09155* + */SSU_RS03100* + genotype was observed in 96.4% (134/139) and 13.6% (3/22) of the pathogenic and commensal pathotypes, respectively (Table 2). Genotypes containing marker *SSU_RS01590* identified fewer pathogenic isolates. Thus, only markers *SSU_RS09525*, *SSU_RS09155*, and *SSU_RS03100* were selected as the ‘best’ predictors of pathotype. The pathogenic and possibly opportunistic isolates possessing these markers belonged to serotype-ST combinations unique to these pathotypes (Additional files 5 and 6). The exceptions are the three commensal isolates possessing these markers which belonged to serotype 3 ST94, serotype 3 ST108, and serotype 8 ST87. To investigate if these three markers were present in Eurasian strains, we further tested the markers in eight well-characterized and highly cited Eurasian references. The markers were tested by BLASTn and identified in all eight reference strains with ≥ 96% identity and 100% coverage (Table 3).

**Table 1:** **Shared candidate VAGs identified by two LASSO analyses of *S. suis*** **genomes**

Pan-gene group* in analysis of 208 genomes	Pan-gene group* in analysis of 161 genomes	*S. suis* strain reference (non-redundant protein WP_ accession)	Size (AA)	Annotation
Group_878	Group_693	SSU_RS09525 (P1/7) (WP_012027987.1)	137	RNA-binding protein
Group_766	Group_584	SSU_RS09155 (P1/7) (WP_012028544.1)	219	Hypothetical protein
Group_1486	Group_1385	SSU_RS03100 (P1/7) (WP_012775033.1)	104	Hypothetical protein
Group_790	Group_739	SSU_RS01590 (P1/7) (WP_012774960.1)	175	Membrane protein/ECF transporter S component

^*^Group name is unique and determined by each ROARY analysis. Hence, group names are different for the same pan-gene cluster

**Table 2:** **The four candidate VAGs and proposed genotype for the three pathotypes of *S. suis* identified by LASSO**

Candidate VAG(s)	No. containing the candidate VAG(s)	Pathogenic (*n* = 139)		Possibly Opportunistic (*n* = 47)		Commensal (*n* = 22)	
		No.	Proportion*	No.	Proportion*	No.	Proportion*
SSU_RS09525	189	138	0.730	42	0.222	9	0.048
SSU_RS09155	178	137	0.770	34	0.191	7	0.039
SSU_RS03100	172	134	0.779	34	0.198	4	0.023
SSU_RS01590	167	127	0.760	31	0.186	9	0.054
SSU_RS09525 SSU_RS09155 SSU_RS03100	168	134	0.798	31	0.185	3	0.018

^*^Positive isolates in the pathotype divided by the number of isolates containing the candidate VAG(s)

**Table 3:** **Presence of novel candidate VAGs in virulent Eurasian *S. suis*** **strains**

Strain	Serotype	ST	% identity			Source	Origin	Accession no.	Refs.
			SSU_RS09155	SSU_RS09525	SSU_RS03100				
GZ1	2	1	96.1	98.8	100	Human	China	CP000837	[74]
SC84	2	7	96.1	98.8	100	Human	China	FM252031	[67]
P1/7	2	1	96.1	98.8	100	Diseased pig	United Kingdom	AM946016	[67]
S735	2	1	96.1	98.8	100	Diseased pig	The Netherlands	CP003736	[75]
ZY05719	2	7	96.1	98.8	100	Diseased pig	China	CP007497	[76]
SC19	2	7	96.1	98.8	99.7	Diseased pig	China	CP020863	[77]
05ZYH33	2	7	96.1	97.4	100	Human	China	CP000407	[25]
10	2	1	96.1	98.8	100	Healthy pig	The Netherlands	CP058742	[78, 79]

### Identification of ARGs

**Figure 4:**
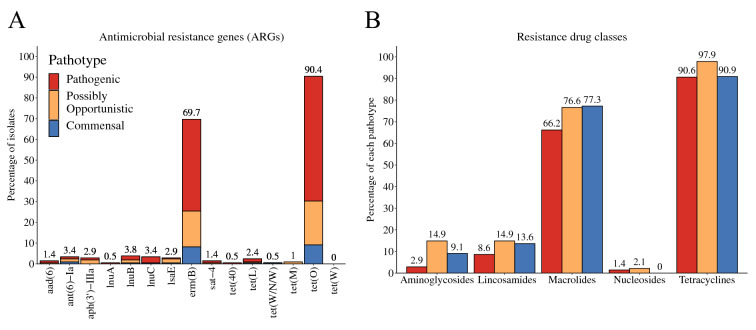
**Antimicrobial resistance genes (ARGs) and drug classes determined in *S. suis* isolates (*n*** = **208).** **A** Percentage of isolates possessing each of the 15 ARGs identified. ARGs were subdivided by pathotype (stacked bar sections; pathogenic, possibly opportunistic, and commensal). **B** Percentage of each pathotype possessing ARGs conferring resistance to each of the five drug classes aminoglycosides, lincosamides, macrolides, nucleosides, and tetracyclines.

The presence of ARGs was predicted in all *S. suis* draft assemblies of the training set (Additional file 7). Fifteen ARGs representing five drug classes (aminoglycoside, lincosamide, macrolide, nucleoside, and tetracycline) were identified in at least one isolate (Figure 4A). The predominant ARGs were *tet*(*O*) (90.4%, 188/208) and *erm*(*B*) (69.7%, 145/208), which confer resistance to tetracycline and MLS (macrolide/lincosamide/streptogramin) antibiotics, respectively. Notably, ~77% of the commensal and possibly opportunistic pathotypes possessed macrolide resistance genes and 14.9% of the possibly opportunistic pathotype possessed aminoglycoside resistance genes compared to 66.2% and 2.9% of the pathogenic pathotype, respectively (Figure 4B). Multidrug resistance (ARGs conferring resistance to ≥ 3 drug classes) was predicted in 11.0% (23/208) of isolates.

### Identification of *S. suis* MGEs and MGE-associated ARGs and VAGs

**Figure 5:**
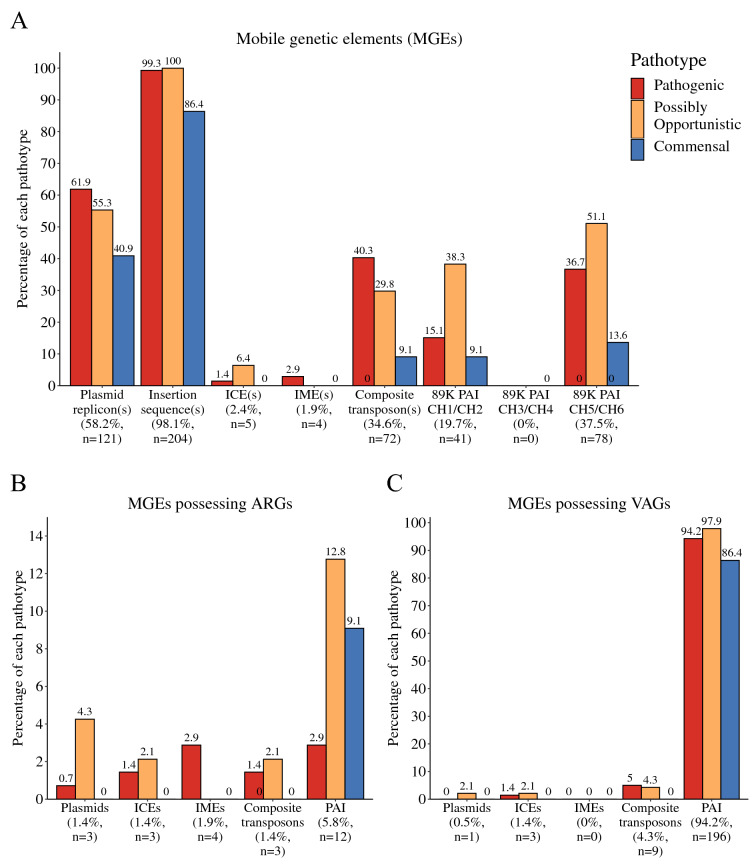
**Mobile genetic elements carrying ARGs and VAGs.** Percentage of each pathotype (pathogenic, possibly opportunistic, and commensal) possessing **A** various MGE types, **B** ARG-associated MGEs, and **C** VAG-associated MGEs. *S. suis* strain 05ZYH33 89 K PAI sequences CH1/CH2 (5′ flanking region), CH3/CH4 (716 bp internal fragment), CH5/CH6 (3′ flanking region). Total percentage and number of isolates possessing each type of MGE are listed under its respective name.

The presence of various MGE types was determined in-silico. Plasmid replicons were predicted in 58.2% (121/208) of *S. suis* genomes via the reference-based method (PlasmidFinder) (Figure 5A), and 15.7% (19/121) of these contained multiple (2–3) plasmid replicons. Ten different plasmids were predicted, and the predominant plasmid types were the *S. suis* plasmids pNSUI060a (54.3%, 113/208), pISU2614 (6.7%, 14/208), and pSSU1 (AB019522) (6.7%, 14/208) (Additional file 7). There was no association (*p*-value > 0.05) between the presence of plasmids and pathotype; however, a majority (61.9%, 86/139) of the pathogenic pathotype contained at least one plasmid compared to a minority (40.9%, 9/22) of the commensal pathotype. Eighty isolates (pathogenic, *n* = 47; possibly opportunistic, *n* = 21; commensal, *n* = 12) lacking plasmids through the reference-based method possessed potentially novel plasmids (Additional file 7). Sequence analysis demonstrated multiple different plasmid sequences. Insertion sequences, ICEs, IMEs, and composite transposons were predicted in 98.1% (204/208), 2.4% (5/208), 1.9% (4/208), and 34.6% (72/208) of genomes, respectively. There was a significant association (*p*-value < 0.05) between the presence of predicted composite transposons and pathotype with 40.3% (56/139) of the pathogenic pathotype possessing at least one composite transposon compared to 9.1% (2/22) of the commensal pathotype. The *S. suis* strain 05ZYH33 89 K PAI was determined by the CH3/CH4 internal PAI sequence, which was lacking in all isolates of the training set (Figure 5A). The CH1/CH2 (5′ flanking region) and CH5/CH6 (3′ flanking region) sequences were identified in 19.7% (41/208) and 37.5% (78/208) of isolates, respectively (see Additional file 8 for sequence alignments). Phages were identified in all but one genome, however only 25.0% (52/208) of isolates possessed intact phages (Additional file 9). The intact phages of 12.5% (26/208) of isolates were predicted to be located in the bacterial chromosome, while the location was unclear for the remaining isolates. No intact phages appeared to be located on plasmids.

The ARGs and VAGs carried on the predicted MGEs were identified by a BLAST search of the MGE draft sequences to the custom ARG-VAG database (Additional file 7). In the training set, 5.8% (12/208) of the isolates contained the ARGs aminoglycoside 6-adenylyltansferase (*SSU05_0957*) and tetracycline resistance protein (*SSU05_0922*) in the *S. suis* strain 05ZYH33 89 K PAI (Figure 5B). Higher percentages of possibly opportunistic (12.8%, 6/47) and commensal (9.1%, 2/22) isolates carried ARGs compared to pathogenic isolates (2.9%, 4/139). ARGs (*erm*(*B*), *lnuC*, and/or *tet*(*M*)) were identified in plasmids, ICEs, and composite transposons of 1.4% (3/208) of isolates and in IMEs of 1.9% (4/208) of isolates. None of the isolates possessing these ARG-associated MGEs were classified as the commensal pathotype.

Putative VAGs were identified on PAI-like sequences of 94.2% (196/208) of isolates (Figure 5C). These VAGs included the *S. suis* strain 05ZYH33 agglutinin receptor (*SSU05_0965*) and type IV secretory system VirB4/VirD4 components (*SSU05_0969*/*SSU05_0973*), which were present in ≥ 75% (≥ 157/208) of genomes (Additional file 7). Other PAI-associated VAGs present in the genomes were NisK/NisR (*SSU05_0906*/*SSU05_0907*), putative zeta toxin (*SSU05_0936*), and putative abortive infection protein (*SSU05_0966*). These PAI-associated VAGs were identified in all three pathotypes with no major difference in distribution by pathotype. VAGs were identified in less than 5% (*n* < 10) of isolates with predicted plasmids, ICEs, IMEs, and transposons, and included *S. suis orf207*, *revS*, and *traG*. Multiple beneficial genes were determined from the MGE drafts (as determined by BLAST and prokka annotations) (Additional file 10). A putative zeta/pezT toxin (*SSU05_0936*) was predominantly identified in the pathogenic and possibly opportunistic pathotypes (70–72%). Many of the MGE drafts in our study contained genes encoding major carbohydrate transport systems (PTS transporters) (28%), a mobility protein (methyl-accepting chemotaxis protein) (72%), and metal resistance proteins (arsenic, calcium) (76–77%). The proposed novel VAGs *SSU_RS09525*, *SSU_RS09155*, and *SSU_RS03100* were not associated with MGEs predicted in this study, as determined by BLAST. In summary, ARGs and VAGs were identified in multiple MGE types and PAI-like regions were the most diverse.

## Discussion

In this study, comparative analysis of 208 previously characterized *S. suis* isolates [31] was performed to gain insights into the distribution and function of the *S. suis* core and accessory genes. Functional comparisons illustrated differences in COG classes with the potential enrichment of virulence-associated genes in the accessory genome. Markers *SSU_RS09525*, *SSU_RS09155*, and *SSU_RS03100* demonstrated strong associations with the pathogenic pathotype presenting novel candidate VAGs for identifying pathogenic *S. suis* strains in North America (predominantly the United States). We investigated the distribution of MGEs and determined that MGEs have the potential to spread resistance genes and putative VAGs.

Functional annotation of the pan-genome was performed using the COG protein database to investigate differences in the abundance of classes between core and accessory genes. Genes involved in nucleotide transport, translation, post-translation modifications, and signal transduction mechanisms (COG F, J, O, and T) were over-represented among the core genes. These functions (represented by COG F, J, O, and T) can more broadly be described as cellular processing and signaling, information storage, and metabolism and are responsible for basic cell function (“housekeeping”) [52, 53]. Accessory genes were more likely to be involved in defense mechanisms and the mobilome (COG X and V), which are functions associated with host- and environmental-interactions, horizontal gene transfer, and niche-adaptation in bacterial pathogens [53–55]. Thus, the distribution and function of the *S. suis* pan-genome support our hypothesis that the accessory genome would be enriched for genes linked to pathogenicity.

Candidate novel VAGs in the *S. suis* accessory genome were selected using chi-square tests and LASSO regression models testing associations between accessory genes and pathotype. Three pan-genes corresponding to *S. suis* strain P1/7 genes *SSU_RS09525*, *SSU_RS09155*, and *SSU_RS03100* were selected as the “best” indicators of pathogenicity for isolates in our study. In our previous study, we demonstrated that a genotype consisting of classical *S. suis* VAGs extracellular protein factor, muramidase-released protein, and suilysin (*epf* + /*mrp* + /*sly* +) only identified 14% of the pathogenic pathotype, while a novel proposed genotype of published VAGs *ofs* and *srtF* (*ofs* + /*srtF* +) was able to identify 95% [39]. Yet, virulent strains lacking *ofs* and/or *srtF* have been reported in North America, indicating these VAGs may not be essential for virulence [3, 56]. The *SSU_RS09525* + */SSU_RS09155* + */SSU_RS03100* + genotype identified 96% of the pathogenic pathotype and only 14% of the commensal pathotype. Traditionally, Eurasian and North American strains are genotypically and phenotypically different and may possess different virulence markers [1, 56]. However, the identification of the *SSU_RS09525*, *SSU_RS09155*, and *SSU_RS03100* markers in all eight Eurasian strains (serotype 2 ST1 or ST7) tested suggests these markers can potentially be applied globally. The five serotype 2 ST1 pathogenic strains in the training set also possessed these three virulence markers. The application of these virulence markers to pathotype Eurasian strains needs to be verified using a large collection of isolates including non-serotype 2 and commensal strains.

Using a genomic approach, the predicted function of these candidate VAGs and their potential relevance to *S. suis* disease were investigated. *SSU_RS09155* and *SSU_RS03100* were annotated as hypothetical proteins and have been reported in virulent *S. suis* strains such as P1/7, SC84, and GZ1. Markers *SSU_RS09155* and *SSU_RS03100* could not be further characterized by COG or by searching the NCBI protein databases, reinforcing that much of the *S. suis* genome is uncharacterized or poorly characterized. *SSU_RS09525* was annotated as an RNA-binding protein (RBP), which is involved in post-transcriptional regulation via regulation of translation initiation, stability, and transcript elongation [57, 58]. RBPs are well-studied in *E. coli* and *Salmonella enterica* serovar Typhimurium and were shown to affect virulence gene expression [59, 60]. *SSU_RS01590* encodes a putative energy-coupling factor transporter substrate-binding protein (ECF transporter S component). ECF transporters are responsible for vitamin uptake and are essential for growth and survival, contributing to the virulence of various gram-positive bacterial pathogens [61, 62]. The strong associations with the pathogenic pathotype and potential virulence-related functions suggest that the proposed markers contribute to the pathogenicity of *S. suis*. The possibility of another primary pathogen and the presence of opportunistic *S. suis* strains will continue to be a concern when studying *S. suis*-associated diseases. However, the pathotype classifications used in this study are what can be currently accomplished given the history of the farms and the diagnostic reports.

Fifteen ARGs with predicted resistance to aminoglycoside, lincosamide, macrolide, nucleoside, and tetracycline antibiotics were identified in the draft genomes of the training set. High predicted resistance to tetracyclines (93%, predominantly *tet*(*O*)) and erythromycin (70%, *erm*(*B*)) was observed similar to previous reports of resistance genes in *S. suis* in North America, Asia, and Europe [63, 64]. Previous studies indicate a higher prevalence of antibiotic resistance among commensal strains, which may act as reservoirs for resistance genes [65, 66]. There was no major difference in the distribution of resistance genes by pathotype, but the commensal and possibly opportunistic pathotypes did tend to have more resistance genes. However, we are aware that genotypic resistance does not guarantee phenotypic resistance. Point mutations are also important resistance mediators and may be further investigated.

Horizontal gene transfer of MGEs is one mechanism by which *S. suis* acquires and spreads resistance genes and putative VAGs. Thus, a reference-based in-silico approach was used for a preliminary investigation of MGEs in the *S. suis* genomes and of genes carried on these elements. Resistance MGEs were identified in only 6% of isolates, largely classified as pathogenic and possibly opportunistic (83%) and were predominantly present in PAI-like regions. However, only a few isolates (3.4%, *n* = 7) had multiple resistance MGEs. ICE and IME are commonly found in *Streptococcus* genomes and play a major role in the dissemination of ARGs in *S. suis* [29, 67]. In our study, only the 69 kb *S. suis* ICESsuZJ20091101-1-like ICEs (*n* = 3) and 1.7 kb *Streptococcus agalactiae* MTnSag1-like IMEs (*n* = 4) carried resistance genes (*erm*(*B*), *lnu*C, and *tet*(*O*)). Less than 2% of isolates possessed resistance plasmids although this may not be uncommon as there are limited reports of *S. suis* plasmids carrying ARGs [28, 29] and resistance plasmids are largely found in gram-negative bacteria [68]. Overall, this MGE mechanism of antimicrobial resistance represents 77%, 2%, and 5% of the predicted aminoglycoside, tetracycline, and lincosamide/macrolide resistance, respectively, regardless of pathotype. Our preliminary findings suggest MGEs continue to play a role, although limited, in the spread of antimicrobial resistance in *S. suis*.

Putative VAGs were identified among the various MGE types, but mostly on PAI-like sequences, and the predominant VAGs encode agglutinin receptor and type IV secretion system components (T4SS). Agglutinin receptors (adhesion proteins) and T4SS contribute to *S. suis* virulence (serotypes 9 and 2, respectively) by promoting anti-phagocytic activity and the release of proinflammatory cytokines [69, 70]. The agglutinin receptor was present in 86% of the commensal pathotype compared to 72% of the pathogenic pathotype while the T4SS components were widely distributed among the three pathotypes (86–96%). Our results indicated a lack of correlation between the presence of MGE-associated VAGs and pathotype. The presence of VAGs in a majority of the commensal pathotype provides further evidence that commensal strains may act as gene reservoirs [66, 71]. Although the 89 K PAI (a *S. suis* MGE) was absent in all isolates in this study, the in silico detection of 89 K PAI sequences (CH1/CH2 and CH5/CH6) and the presence of PAI VAGs suggest some genetic similarity between North American isolates and the virulent *S. suis* strain 05ZYH33 [45]. Together our preliminary findings agree with publications stating that MGEs carry a range of genes that contribute to the survival and adaptation of pathogenic bacteria to dynamic environments [22, 72]. Moreover, these results suggest MGE-mediated transfer of genes is possible in North American *S. suis* isolates.

Comparative genomic analysis of 208 *S. suis* isolates demonstrated a potential enrichment of virulence-associated genes in the accessory genome and elucidated a novel VAG genotyping scheme (*SSU_RS09525* + */SSU_RS09155* + */SSU_RS03100* +) for identifying pathogenic *S. suis* strains in North America. We further described preliminary data on the diversity of MGEs in the training set and determined that MGEs have the potential to spread resistance genes and putative VAGs between *S. suis* strains. Further research is needed in vitro to evaluate the contribution of the proposed VAGs to virulence.

## Supplementary Information

**Additional file 1**: **Genome assembly and annotation statistics of *S. suis* isolates (*n*** = **208).** Genome assembly and annotation statistics, such as predicted genome length, number of contigs, GC %, average genome coverage, and number of protein coding sequences, for each of the 208 *S. suis* isolates in the training set.

**Additional file 2: Gene clusters (*n* = 8231) identified in Roary analysis of *S. suis* genomes (*n*** = **208).** Binary matrix representing the presence (1) and absence (0) of all 8231 gene clusters identified by the Roary analysis performed on all 208 *S. suis* genomes.

**Additional file 3: COG functional classifications for the 995 pan-gene clusters that met the criteria.** Results of the BLAST searches against the Predicted Cluster of Orthologous Groups (COG) database. Classification as core or accessory and present (yes) or absent (no) in each pathotype was provided for each pan-gene cluster.

**Additional file 4:** **Gene clusters (*n* = 6407) identified in Roary analysis of *S. suis* genomes (*n*** = **161).** Binary matrix representing the presence (1) and absence (0) of all 6407 gene clusters identified by the Roary analysis performed on the 161 *S. suis* genomes representing only the pathogenic and commensal pathotypes.

**Additional file 5: Novel candidate VAGs in S. suis serotypes and STs**. Serotype-ST combinations of *S. suis* possessing the *SSU_RS09525*, *SSU_RS09155*, and *SSU_RS03100* markers.

**Additional file 6: Presence-absence of novel candidate VAGs in *S. suis* serotypes and STs.** Binary matrix representing the presence (1) or absence (0) of the *SSU_RS09525*, *SSU_RS09155*, and *SSU_RS03100* markers in the training set representing 20 serotypes and 58 STs.

**Additional file 7:** **ARGs, VAGs, and MGEs identified in *S. suis* genomes (*n*** = **208).** Binary matrix representing the presence (1/yes) and absence (0/no) of ARGs, MGEs, MGE-associated ARGs, and MGE-associated VAGs.

**Additional file 8:** **Sequence alignments of 89 K PAI CH1/CH2 and CH5/CH6.** Alignments of the CH1/CH2 and CH5/CH6 DNA sequences using the *S. suis* strain 05ZYH33 as the reference and generated using Boxshade.

**Additional file 9**: **Identification and predicted location of phages in *S. suis* genomes (*n*** = **208).** List of intact, questionable, and incomplete phages identified by PHASTER and their location (chromosome, plasmid) as predicted by viralVerify.

**Additional file 10**: **Prokka annotations of MGE drafts.** List of prokka annotations of the plasmid, ICE, IME, composite transposon, and 89 K PAI draft sequences identified in this study.

## Data Availability

The dataset analyzed during the current study is available in the Sequence Read Archive database: accession numbers SRR9123061-SRR9123268. The accession numbers are also provided in Additional file 1. Custom scripts utilized for the functional annotation and statistical analyses are available through GoogleDrive [73].

## Abbreviations

ARGs: antimicrobial resistance genes
COG: Clusters of Orthologous Groups of proteins
ICE: integrative conjugative elements
IME: integrative mobilizable elements
LASSO: Least Absolute Shrinkage and Selection Operator
MLST/ST: multilocus sequence typing
MGEs: mobile genetic elements
PAI: pathogenicity island
VAGs: virulence-associated genes
