# Supplementary material for: Comparative analysis of Streptococcus suis genomes identifies novel candidate virulence-associated genes in North American isolates
Source: Vet Res. 2022 Mar 18;53:23. doi: 10.1186/s13567-022-01039-8 (PMC8932342; doi:10.1186/s13567-022-01039-8)
Supplement: Supplementary file 8 — Additional file 8: Sequence alignments of 89 K PAI CH1/CH2 and CH5/CH6. Alignments of the CH1/CH2 and CH5/CH6 DNA sequences using the S. suis strain 05ZYH33 as the reference and generated using Boxshade. [file 13567_2022_1039_MOESM8_ESM.docx]

**Additional file 8. Sequence alignments of 89K PAI CH1/CH2 and CH5/CH6.**

# **89K PAI CH1/CH2**

05ZYH33 1 CACGCATCTCGTAGAGTTTGACAGGAATGCCACGTTTGGCAATCTGATAAGCAGCTTCTGAGCCAGCCAAGCCAGCTCCTATAACATTAATGTGGGTTTGAGACATGAAACTAATACCTC

2016UMN1917.1 1 .................................................T......................................................................

2016UMN1440.1 1 .............A.................................................................G.....G..................................

2014UMN3714.6 1 ........................................................................................................................

2016UMN146.4 1 .............A.................................................................G.....G..................................

2016UMN668.5 1 ........................................................................................................................

2016UMN1367.1,

2017UMN1574.421, and

2017KSU1528.8 1 ........................................................................................................................

2016UMN2465.63B 1 .............A.................................................................G.....G..................................

2016UMN2500.63 1 ........................................................................................................................

2015UMN592.5 1 .............A.................................................................G.....G..................................

2016UMN1524.93 1 .............A.................................................................G.....G..................................

2017UMN236.4 1 .........................................................................G.....G.....G..................................

2017UMN631.2 1 ........................................................................................................................

2016UMN2813.1 1 ........................................................................................................................

2016UMN3027.6 1 .............A.................................................................G.....G..................................

2014UMN2148.7 1 .............A.................................................................G.....G..................................

2017UMN55.9 1 ........................................................................................................................

2017UMN92.8 1 .............A....................C............................................G.....G..................................

2017UMN144.4 1 ........................................................................................................................

2017UMN145.1 1 .............A....................C............................................G.....G..................................

2017UMN164.7 1 .........................................................................G.....G.....G..................................

2015UMN2795.3 and

2017UMN296.1 1 ........................................................................................................................

2017UMN1161.8 1 .........................................................................G.....G.....G..................................

2015UMN1635.9 1 ........................................................................................................................

2014KSU27.8 1 ...............................................................................G.....G..................................

2014KSU2091.1 1 --.............................G...............................................G........................................

2015KSU2327.2 1 --.............................G...............................................G........................................

2016KSU47.5 1 .........................................................................G.....G.....G..................................

2014UMN3951.1 1 --...........A........G.....A.....C............................................G.....G.....A............................

2015UMN193.3 1 .........................................................................G.....G.....G..................................

2015UMN370.6 1 ..................................C.................C..........................G.....G.....A............................

2015UMN64.3 1 ..................................C.................G..........................G.....G..................................

2015UMN1735.7 1 .........................................................................G.....G.....G..................................

2014UMN3624.74 1 ........................................................................................................................

2014UMN3720.5 1 .............A.................................................................G.....G..................................

2014UMN3930.3 1 ........................................................................................................................

2014UMN3624.71 1 ........................................................................................................................

2015UMN865.5 1 .............A.................................................................G.....G..................................

2015UMN4053.5 1 ........................................................................................................................

05ZYH33 121 TAGCCAGAATATCTGACTACTGTTTGGGAATACCCAAAAACAGAACCTTTCTAAATTTTATACCTATCTAGTATACCAGAAAATGGCTAGAAAGGACAAAACTACCTTCATTGAAATGAA

2016UMN1917.1 121 ........................................T....................................................A..........................

2016UMN1440.1 121 ...T...............................C.........................................................A.......................A..

2014UMN3714.6 121 ........................................................................................................................

2016UMN146.4 121 ...T...............................C.........................................................A.......................A..

2016UMN668.5 121 ........................................................................................................................

2016UMN1367.1,

2017UMN1574.421, and

2017KSU1528.8 121 ........................................................................................................................

2016UMN2465.63B 121 ...T...............................C.........................................................A.......................A..

2016UMN2500.63 121 ........................................................................................................................

2015UMN592.5 121 ...T...............................C.........................................................A.......................A..

2016UMN1524.93 121 ...T...............................C.........................................................A.......................A..

2017UMN236.4 121 .............................................................................................A..........................

2017UMN631.2 121 ........................................................................................................................

2016UMN2813.1 121 ........................................................................................................................

2016UMN3027.6 121 ...T...............................C.........................................................A.......................A..

2014UMN2148.7 121 ...T...............................C.........................................................A.......................A..

2017UMN55.9 121 ........................................................................................................................

2017UMN92.8 121 ...................................C.........................................................A..........................

2017UMN144.4 121 ........................................................................................................................

2017UMN145.1 121 ...................................C.........................................................A..........................

2017UMN164.7 121 ...................................C.........................................................A..........................

2015UMN2795.3 and

2017UMN296.1 121 ........................................................................................................................

2017UMN1161.8 121 .............................................................................................A..........................

2015UMN1635.9 121 ........................................................................................................................

2014KSU27.8 121 ...................................C.........................................................A..........................

2014KSU2091.1 119 .........G.........................C.........................................................A..........................

2015KSU2327.2 119 .........G.........................C.........................................................A..........................

2016KSU47.5 121 .............................................................................................A..........................

2014UMN3951.1 119 ...................................C.........................................................A..........................

2015UMN193.3 121 .............................................................................................A..........................

2015UMN370.6 121 ...................................C...................A.....................................A..........................

2015UMN64.3 121 .................C.............................................A.............................A..........................

2015UMN1735.7 121 ...................................C.........................................................A..........................

2014UMN3624.74 121 ........................................................................................................................

2014UMN3720.5 121 ...T...............................C.........................................................A.......................A..

2014UMN3930.3 121 ........................................................................................................................

2014UMN3624.71 121 ........................................................................................................................

2015UMN865.5 121 ...T...............................C.........................................................A.......................A..

2015UMN4053.5 121 ........................................................................................................................

05ZYH33 241 AGTAGTTTCACCTTTTTACATAATATATTTTATGTATATCCTTTACTGTTCTATCGCTTATTTACAATAGCTCCGCTATATCTCCAACAACCTCAAAATTCCAACCAAGTCTCCTATAAG

2016UMN1917.1 241 .......C.G.T................................G......A.............................................................T......

2016UMN1440.1 241 .......................................T................................................................................

2014UMN3714.6 241 ........................................................................................................................

2016UMN146.4 241 .......................................T................................................................................

2016UMN668.5 241 ........................................................................................................................

2016UMN1367.1,

2017UMN1574.421, and

2017KSU1528.8 241 ........................................................................................................................

2016UMN2465.63B 241 .......................................T................................................................................

2016UMN2500.63 241 ........................................................................................................................

2015UMN592.5 241 .......................................T................................................................................

2016UMN1524.93 241 .......................................T................................................................................

2017UMN236.4 241 .......C...............................T................A...............................................................

2017UMN631.2 241 ........................................................................................................................

2016UMN2813.1 241 ........................................................................................................................

2016UMN3027.6 241 .......................................T................................................................................

2014UMN2148.7 241 .......................................T................................................................................

2017UMN55.9 241 ........................................................................................................................

2017UMN92.8 241 .......C...T................................G...........................................................................

2017UMN144.4 241 ........................................................................................................................

2017UMN145.1 241 .......C...TG.....................C....T................................................................................

2017UMN164.7 241 .......................................T................................................................................

2015UMN2795.3 and

2017UMN296.1 241 ........................................................................................................................

2017UMN1161.8 241 .......C...............................T................A...............................................................

2015UMN1635.9 241 ........................................................................................................................

2014KSU27.8 241 .......................................T................................................................................

2014KSU2091.1 239 ........................................A...G..A......A.................................................................

2015KSU2327.2 239 ........................................A...G..A......A..........................................G......................

2016KSU47.5 241 .......C...............................T................A...............................................................

2014UMN3951.1 239 .......C....G....G.....................T...............................................T................................

2015UMN193.3 241 .......C...............................T................A...............................................................

2015UMN370.6 241 ........................................................................................................................

2015UMN64.3 241 ....A..................................T....G.............................................................G.............

2015UMN1735.7 241 ............C...........................................................................................................

2014UMN3624.74 241 ........................................................................................................................

2014UMN3720.5 241 .......................................T................................................................................

2014UMN3930.3 241 ........................................................................................................................

2014UMN3624.71 241 ........................................................................................................................

2015UMN865.5 241 .......................................T................................................................................

2015UMN4053.5 241 ........................................................................................................................

05ZYH33 361 AGTCGATTTCTTCCTGACTATACGGGAAAGTATTCAGCAAAATGGAATCCATTCCCAATTCATGAGCAATAGCTATATCGGATGTCAAATCATTACCAACCATAACCGTTTCCTCAACAT

2016UMN1917.1 361 ........................................................................................................................

2016UMN1440.1 361 ..................................................................................................................T.....

2014UMN3714.6 361 ........................................................................................................................

2016UMN146.4 361 ..................................................................................................................T.....

2016UMN668.5 361 ........................................................................................................................

2016UMN1367.1,

2017UMN1574.421, and

2017KSU1528.8 361 ........................................................................................................................

2016UMN2465.63B 361 ..................................................................................................................T.....

2016UMN2500.63 361 ........................................................................................................................

2015UMN592.5 361 ..................................................................................................................T.....

2016UMN1524.93 361 ..................................................................................................................T.....

2017UMN236.4 361 ........................................................................................................................

2017UMN631.2 361 ........................................................................................................................

2016UMN2813.1 361 ........................................................................................................................

2016UMN3027.6 361 ..................................................................................................................T.....

2014UMN2148.7 361 ..................................................................................................................T.....

2017UMN55.9 361 ........................................................................................................................

2017UMN92.8 361 ........................................................................................................................

2017UMN144.4 361 ........................................................................................................................

2017UMN145.1 361 ........................................................................................................................

2017UMN164.7 361 .....................................T..................................................................................

2015UMN2795.3 and

2017UMN296.1 361 ........................................................................................................................

2017UMN1161.8 361 ........................................................................................................................

2015UMN1635.9 361 ........................................................................................................................

2014KSU27.8 361 ........................................................................................................................

2014KSU2091.1 359 ........................................................................................................................

2015KSU2327.2 359 ...................................................................................................T....................

2016KSU47.5 361 ........................................................................................................................

2014UMN3951.1 359 ........................................................................................................................

2015UMN193.3 361 ........................................................................................................................

2015UMN370.6 361 ........................................................................................................................

2015UMN64.3 361 ................................................................................................................T.......

2015UMN1735.7 361 ........................................................................................................................

2014UMN3624.74 361 ........................................................................................................................

2014UMN3720.5 361 ..................................................................................................................T.....

2014UMN3930.3 361 ........................................................................................................................

2014UMN3624.71 361 ........................................................................................................................

2015UMN865.5 361 ..................................................................................................................T.....

2015UMN4053.5 361 ........................................................................................................................

05ZYH33 481 CAAGATTATTTTCCTCTAAAACCAGTTGAAGAAATGCTGGTTCAGGCTTCTTCATCTTAAAGTCAGAAGAGATATAAATTTTTTCCATCAAATCAGCACAACCTGTCTGTTCAATTTCTG

2016UMN1917.1 481 ........................................................................................................................

2016UMN1440.1 481 ...................................................................G....................................................

2014UMN3714.6 481 ........................................................................................................................

2016UMN146.4 481 ...................................................................G....................................................

2016UMN668.5 481 ........................................................................................................................

2016UMN1367.1,

2017UMN1574.421, and

2017KSU1528.8 481 ........................................................................................................................

2016UMN2465.63B 481 ...................................................................G....................................................

2016UMN2500.63 481 ........................................................................................................................

2015UMN592.5 481 ...................................................................G....................................................

2016UMN1524.93 481 ...................................................................G....................................................

2017UMN236.4 481 ........................................................................................................................

2017UMN631.2 481 ........................................................................................................................

2016UMN2813.1 481 ........................................................................................................................

2016UMN3027.6 481 ...................................................................G....................................................

2014UMN2148.7 481 ...................................................................G....................................................

2017UMN55.9 481 ........................................................................................................................

2017UMN92.8 481 ........................................................................................................................

2017UMN144.4 481 ........................................................................................................................

2017UMN145.1 481 ................................................................................................................G.......

2017UMN164.7 481 ........................................................................................................................

2015UMN2795.3 and

2017UMN296.1 481 ........................................................................................................................

2017UMN1161.8 481 ........................................................................................................................

2015UMN1635.9 481 ........................................................................................................................

2014KSU27.8 481 ........................................................................................................................

2014KSU2091.1 479 ..............................................................................................C.........................

2015KSU2327.2 479 ........................................................................................................................

2016KSU47.5 481 ........................................................................................................................

2014UMN3951.1 479 ..............................................................................................C.........................

2015UMN193.3 481 ........................................................................................................................

2015UMN370.6 481 ..................................................................................C..........T..........................

2015UMN64.3 481 .....................G...............C........................................................C.........................

2015UMN1735.7 481 ........................................................................................................................

2014UMN3624.74 481 ........................................................................................................................

2014UMN3720.5 481 ...................................................................G....................................................

2014UMN3930.3 481 ........................................................................................................................

2014UMN3624.71 481 ........................................................................................................................

2015UMN865.5 481 ...................................................................G....................................................

2015UMN4053.5 481 ........................................................................................................................

05ZYH33 601 CCTGAGTAAAAATTCGTTGAGCATTGGAGAGAATAAATAATCGCACTTCTCTGTCCTTTAATGTCTGTAGACTAGTTAGGGTATTTTCATAAGCCTCTAACTTTTCACGTGAAAGCACGC

2016UMN1917.1 601 .........................A..................G..................................A........................................

2016UMN1440.1 601 ............................................G.......A...................................................................

2014UMN3714.6 601 .............C..............................G.......A..T................................................................

2016UMN146.4 601 ............................................G.......A...................................................................

2016UMN668.5 601 ........................................................................................................................

2016UMN1367.1,

2017UMN1574.421, and

2017KSU1528.8 601 ........................................................................................................................

2016UMN2465.63B 601 ............................................G.......A...................................................................

2016UMN2500.63 601 ........................................................................................................................

2015UMN592.5 601 ............................................G.......A...................................................................

2016UMN1524.93 601 ............................................G.......A...................................................................

2017UMN236.4 601 .............C..............................G.......A..T................................................................

2017UMN631.2 601 ........................................................................................................................

2016UMN2813.1 601 ........................................................................................................................

2016UMN3027.6 601 ............................................G.......A...................................................................

2014UMN2148.7 601 ............................................G.......A...................................................................

2017UMN55.9 601 ........................................................................................................................

2017UMN92.8 601 ........................................................................................................................

2017UMN144.4 601 ........................................................................................................................

2017UMN145.1 601 ..................................................................................................................A.....

2017UMN164.7 601 ............................................G.......A..T................................................................

2015UMN2795.3 and

2017UMN296.1 601 ........................................................................................................................

2017UMN1161.8 601 .............C..............................G.......A..T................................................................

2015UMN1635.9 601 ........................................................................................................................

2014KSU27.8 601 ..C.................................................A...................................................................

2014KSU2091.1 599 ............................................G.......A..T........................C.......................................

2015KSU2327.2 599 ............................................G.......A.........................................T....................T....

2016KSU47.5 601 .............C..............................G.......A..T................................................................

2014UMN3951.1 599 ............................................G.......A..T................................................................

2015UMN193.3 601 .............C..............................G.......A..T................................................................

2015UMN370.6 601 ........................................................................................................................

2015UMN64.3 601 ..C.....................................................................................................................

2015UMN1735.7 601 ........................................................................................................................

2014UMN3624.74 601 .............C..............................G.......A..T................................................................

2014UMN3720.5 601 ............................................G.......A...................................................................

2014UMN3930.3 601 ........................................................................................................................

2014UMN3624.71 601 ........................................................................................................................

2015UMN865.5 601 ............................................G.......A...................................................................

2015UMN4053.5 601 ........................................................................................................................

05ZYH33 721 GGAAAATCGTGGCTACTACTTGTCCAAAAGTCTCTAAATCAGTCGGCTTATTGGTACTTTGGTTTTCATTTGGTGCATCCGTTAATAGACGAATGAAGATTGTTTCTAAATCAATTTCGA

2016UMN1917.1 721 ......................C.................................................................................................

2016UMN1440.1 721 ......................C.................................................................G..........G....................

2014UMN3714.6 721 ........................................................................................................................

2016UMN146.4 721 ......................C.................................................................G..........G....................

2016UMN668.5 721 ........................................................................................................................

2016UMN1367.1,

2017UMN1574.421, and

2017KSU1528.8 721 ........................................................................................................................

2016UMN2465.63B 721 ......................C.................................................................G..........G....................

2016UMN2500.63 721 ........................................................................................................................

2015UMN592.5 721 ......................C.................................................................G..........G....................

2016UMN1524.93 721 ......................C.................................................................G..........G....................

2017UMN236.4 721 ........................................................................................................................

2017UMN631.2 721 ........................................................................................................................

2016UMN2813.1 721 ........................................................................................................................

2016UMN3027.6 721 ......................C.................................................................G..........G....................

2014UMN2148.7 721 ......................C.................................................................G..........G....................

2017UMN55.9 721 ........................................................................................................................

2017UMN92.8 721 ........................................................................................................................

2017UMN144.4 721 ........................................................................................................................

2017UMN145.1 721 ......................C.................................................................G.............................A.

2017UMN164.7 721 ........................................................................................................................

2015UMN2795.3 and

2017UMN296.1 721 ........................................................................................................................

2017UMN1161.8 721 ........................................................................................................................

2015UMN1635.9 721 ........................................................................................................................

2014KSU27.8 721 ......................C.................................................................G...............................

2014KSU2091.1 719 ........................................................................................................................

2015KSU2327.2 719 .........C....................................T.........................................G........T....................A.

2016KSU47.5 721 ........................................................................................................................

2014UMN3951.1 719 ........................................................................................................................

2015UMN193.3 721 ........................................................................................................................

2015UMN370.6 721 ..........A...........C.............................A............G......................G...............................

2015UMN64.3 721 ......................C......................................A.............T............G...............................

2015UMN1735.7 721 ........................................................................................G...............................

2014UMN3624.74 721 ........................................................................................................................

2014UMN3720.5 721 ......................C.................................................................G..........G....................

2014UMN3930.3 721 ........................................................................................................................

2014UMN3624.71 721 ........................................................................................................................

2015UMN865.5 721 ......................C.................................................................G..........G....................

2015UMN4053.5 721 ........................................................................................................................

05ZYH33 841 CATACTGATAACTCACTGTTTCCGCGAGAGAGCTCTCTGCCTCTTCTACAAAACGATGATAGGCATTTTTCAACTGGCGCGGTCGATAGGAACAACCAAAAGCATTGTAAATCTGTGTTA

2016UMN1917.1 841 ........................................................................................................................

2016UMN1440.1 841 .............................A.T........................................................................................

2014UMN3714.6 841 .....CT.................................................................................................................

2016UMN146.4 841 .............................A.T........................................................................................

2016UMN668.5 841 ........................................................................................................................

2016UMN1367.1,

2017UMN1574.421, and

2017KSU1528.8 841 ........................................................................................................................

2016UMN2465.63B 841 .............................A.T........................................................................................

2016UMN2500.63 841 ........................................................................................................................

2015UMN592.5 841 .............................A.T........................................................................................

2016UMN1524.93 841 .............................A.T........................................................................................

2017UMN236.4 841 .....CT.................................................................................................................

2017UMN631.2 841 ........................................................................................................................

2016UMN2813.1 841 ........................................................................................................................

2016UMN3027.6 841 .............................A.T........................................................................................

2014UMN2148.7 841 .............................A.T........................................................................................

2017UMN55.9 841 ........................................................................................................................

2017UMN92.8 841 .....C...................A......................................................A.......................................

2017UMN144.4 841 ........................................................................................................................

2017UMN145.1 841 ...............................T................T..........................................G............................

2017UMN164.7 841 ...............................T........................................................................................

2015UMN2795.3 and

2017UMN296.1 841 ........................................................................................................................

2017UMN1161.8 841 .....CT.................................................................................................................

2015UMN1635.9 841 .....................................................................................................T..................

2014KSU27.8 841 ...............................C........................................................................................

2014KSU2091.1 839 ........................................................................................................................

2015KSU2327.2 839 ...............................T.....C.........................................T........................................

2016KSU47.5 841 .....CT.................................................................................................................

2014UMN3951.1 839 .............................T..........................................................................................

2015UMN193.3 841 .....CT.................................................................................................................

2015UMN370.6 841 ....................G..........T........................................................................................

2015UMN64.3 841 ..............................CT........................................................................................

2015UMN1735.7 841 ...............................T........................................................................................

2014UMN3624.74 841 .....CT.................................................................................................................

2014UMN3720.5 841 .............................A.T........................................................................................

2014UMN3930.3 841 ........................................................................................................................

2014UMN3624.71 841 ........................................................................................................................

2015UMN865.5 841 .............................A.T........................................................................................

2015UMN4053.5 841 ........................................................................................................................

05ZYH33 961 GCTGATTCCAGACCTCTAGTTTATTCTCATCTGTACGAATATCAACCAATGTACCATAAAAATCGAAGATATAGTTTTTGTAACTTTTCATGTCTTTCTCCTAATCTTTAGACATCATTT

2016UMN1917.1 961 ..................................................................................................................G.....

2016UMN1440.1 961 ........................................................................................................................

2014UMN3714.6 961 ........................................................................................................................

2016UMN146.4 961 ........................................................................................................................

2016UMN668.5 961 ........................................................................................................................

2016UMN1367.1,

2017UMN1574.421, and

2017KSU1528.8 961 ........................................................................................................................

2016UMN2465.63B 961 ........................................................................................................................

2016UMN2500.63 961 ........................................................................................................................

2015UMN592.5 961 ........................................................................................................................

2016UMN1524.93 961 ........................................................................................................................

2017UMN236.4 961 ........................................................................................................................

2017UMN631.2 961 ........................................................................................................................

2016UMN2813.1 961 ........................................................................................................................

2016UMN3027.6 961 ........................................................................................................................

2014UMN2148.7 961 ........................................................................................................................

2017UMN55.9 961 ........................................................................................................................

2017UMN92.8 961 ..................................................................................................................G.....

2017UMN144.4 961 ........................................................................................................................

2017UMN145.1 961 ..................................................................................................................G.....

2017UMN164.7 961 ........................................................................................................................

2015UMN2795.3 and

2017UMN296.1 961 ........................................................................................................................

2017UMN1161.8 961 ........................................................................................................................

2015UMN1635.9 961 ........................................................................................................................

2014KSU27.8 961 ..................................................................................................................G.....

2014KSU2091.1 959 ....................................A.............................................................................G.....

2015KSU2327.2 959 .........................T........................................................................................G.....

2016KSU47.5 961 ........................................................................................................................

2014UMN3951.1 959 ..................................................................................................................G.....

2015UMN193.3 961 ........................................................................................................................

2015UMN370.6 961 ...........G.............T........................................................................................G.....

2015UMN64.3 961 ........................................G.............................................G..............C............G.....

2015UMN1735.7 961 ..................................................................................................................G.....

2014UMN3624.74 961 ........................................................................................................................

2014UMN3720.5 961 ........................................................................................................................

2014UMN3930.3 961 ........................................................................................................................

2014UMN3624.71 961 ........................................................................................................................

2015UMN865.5 961 ........................................................................................................................

2015UMN4053.5 961 ........................................................................................................................

05ZYH33 1081 TTTATATTATACCCCACAACGAAAACGTTTGCAACAATAGAACGAAAAAACCTACCACATTAAGTGATAGGTTTTTATTTTGATTGGAAACTCTATTATTTAAGAGTAACAATTGCACAA

2016UMN1917.1 1081 ..............................................................................................G.........................

2016UMN1440.1 1081 ........................................................................................................................

2014UMN3714.6 1081 ........................................................................................................................

2016UMN146.4 1081 ...............................................................................................................G........

2016UMN668.5 1081 ........................................................................................................................

2016UMN1367.1,

2017UMN1574.421, and

2017KSU1528.8 1081 ........................................................................................................................

2016UMN2465.63B 1081 ...............................................................................................................R........

2016UMN2500.63 1081 ........................................................................................................................

2015UMN592.5 1081 ...............................................................................................................R........

2016UMN1524.93 1081 ........................................................................................................................

2017UMN236.4 1081 ........................................................................................................................

2017UMN631.2 1081 ........................................................................................................................

2016UMN2813.1 1081 ........................................................................................................................

2016UMN3027.6 1081 ...............................................................................................................R........

2014UMN2148.7 1081 ........................................................................................................................

2017UMN55.9 1081 ........................................................................................................................

2017UMN92.8 1081 .............T.......................C.....A............................................................................

2017UMN144.4 1081 ........................................................................................................................

2017UMN145.1 1081 ........................................................................................................................

2017UMN164.7 1081 ........................................................................................................................

2015UMN2795.3 and

2017UMN296.1 1081 ........................................................................................................................

2017UMN1161.8 1081 ........................................................................................................................

2015UMN1635.9 1081 ........................................................................................................................

2014KSU27.8 1081 ...........................................A............................................................................

2014KSU2091.1 1079 ........................................................................................................................

2015KSU2327.2 1079 ........................................................................................................................

2016KSU47.5 1081 ........................................................................................................................

2014UMN3951.1 1079 ........................................................................................................................

2015UMN193.3 1081 ........................................................................................................................

2015UMN370.6 1081 .............T....................T........A................C.G.........................................................

2015UMN64.3 1081 ..................................T........A................C.G.........................................................

2015UMN1735.7 1081 ........................................................................................................................

2014UMN3624.74 1081 ........................................................................................................................

2014UMN3720.5 1081 ...............................................................................................................R........

2014UMN3930.3 1081 ........................................................................................................................

2014UMN3624.71 1081 ........................................................................................................................

2015UMN865.5 1081 ...............................................................................................................R........

2015UMN4053.5 1081 ........................................................................................................................

05ZYH33 1201 CCTGTTAAATGTGCAGTATCAAGGGGTTTGAGTTCTTAATTTGAAGTTTGGCAGGAATTTTGGCATAAAATTGAATCCAACCCTGCACTTTTAATATTTTGTGTGATTTCTTCAGAAACT

2016UMN1917.1 1201 ................................................................................T...........T...........................

2016UMN1440.1 1201 ................................................................................T.......................A...............

2014UMN3714.6 1201 ...............................................C................................T.......................................

2016UMN146.4 1201 ...............................................C................................T.......................................

2016UMN668.5 1201 ................................................................................T....................A..................

2016UMN1367.1,

2017UMN1574.421, and

2017KSU1528.8 1201 ...............................................C................................T.......................................

2016UMN2465.63B 1201 ......G.........................................................................T....................A..................

2016UMN2500.63 1201 ...............................................C................................T.......................................

2015UMN592.5 1201 ................................................................................T....................A..................

2016UMN1524.93 1201 .A.............A................................................................T...........G...........................

2017UMN236.4 1201 .A.............A................................................................T...........G...........................

2017UMN631.2 1201 ...............................................C................................T.......................................

2016UMN2813.1 1201 ...............................................C................................T.......................................

2016UMN3027.6 1201 ...............................................C................................T.......................................

2014UMN2148.7 1201 ................................................................................T.......................A...............

2017UMN55.9 1201 ...............................................C................................T.......................................

2017UMN92.8 1201 ........................A............G..........................................T.......................................

2017UMN144.4 1201 ...............................................C................................T.......................................

2017UMN145.1 1201 ................................................................................T.......................A...............

2017UMN164.7 1201 ...............................................C................................T.......................................

2015UMN2795.3 and

2017UMN296.1 1201 ................................................................................T...........T...........................

2017UMN1161.8 1201 ...............................................C............T..................T.......................................

2015UMN1635.9 1201 ...............................................................................GT.......................................

2014KSU27.8 1201 .............................T..................................................T...........T...........................

2014KSU2091.1 1199 ...............................................C................................T.......................................

2015KSU2327.2 1199 ...............................................C................................T.......................................

2016KSU47.5 1201 ...............A................................................................T...........G...........................

2014UMN3951.1 1199 ................................................................................T....................A..................

2015UMN193.3 1201 ...............A................................................................T...........G.........C.................

2015UMN370.6 1201 ................................................................................T.......................................

2015UMN64.3 1201 ........................................................................................................................

2015UMN1735.7 1201 ...............................................C................................T.......................................

2014UMN3624.74 1201 ...............................................C................................T.......................................

2014UMN3720.5 1201 ................................................................................T....................A..................

2014UMN3930.3 1201 ...............................................C................................T.......................................

2014UMN3624.71 1201 ...............................................C................................T.......................................

2015UMN865.5 1201 ................................................................................T....................A..................

2015UMN4053.5 1201 ...............................................C................................T.......................................

05ZYH33 1321 GTTTTATCTAAAGCATTAAACCAATGACTATATGTGTTCAAAGTTGTTGATTTATCAGCATGCCCCATTCGTCTTGCTACATATAAAATATCTTTTTTCAAAACATTTATTAGATAAGAA

2016UMN1917.1 1321 ..............................................................T..............C..........................................

2016UMN1440.1 1321 .......................G...........A....................G.....T.....A.....G..........................G.....G........----

2014UMN3714.6 1321 .......................G...........A....................G.....T.....A.....G..........................G.....G............

2016UMN146.4 1321 .......................G...........A....................G.....T.....A.....G.........................................----

2016UMN668.5 1321 .......................G.....G.....A....................G.....T.....A.....G..........................G.....G........G..T

2016UMN1367.1,

2017UMN1574.421, and

2017KSU1528.8 1321 .......................G...........A....................G.....T.....A.....G..........................G.....G............

2016UMN2465.63B 1321 .......................G.....G.....A....................G.....T.....A.....G..........................G.........---------

2016UMN2500.63 1321 .......................G...........A....................G.....T.....A.....G..........................G.....G...........-

2015UMN592.5 1321 .......................G.....G.....A....................G.....T.....A.....G..........................G.....G............

2016UMN1524.93 1321 .......................G.....G..A..A....................G.....T.....A.....G................-----------------------------

2017UMN236.4 1321 .......................G.....G..A..A....................G.....T.....A.....G...............................--------------

2017UMN631.2 1321 .......................G...........A....................G.....T.....A.....G..........................G.....G........G..T

2016UMN2813.1 1321 .......................G...........A....................G.....T.....A.....G..........................G.....G............

2016UMN3027.6 1321 .......................G...........A....................G.....T.....A.....G..........................G.....G........G..T

2014UMN2148.7 1321 .......................G...........A....................G.....T.....A.....G..........................G.....G........----

2017UMN55.9 1321 .......................G...........A....................G.....T.....A.....G..........................G.....G............

2017UMN92.8 1321 .......................G...........A....................G.....T.....A.....G..........................G.....G............

2017UMN144.4 1321 .......................G...........A....................G.....T.....A.....G..........................G.....G............

2017UMN145.1 1321 .......................G...........A....................G.....T.....A.....G..........................G.....G........G..T

2017UMN164.7 1321 .......................G...........A....................G.....T.....A.....G..........................G.....G...........T

2015UMN2795.3 and

2017UMN296.1 1321 ..............................................................T..............C..........................................

2017UMN1161.8 1321 .......................G...........A....................G.....T.....A.....G..........................G.....G........G..T

2015UMN1635.9 1321 ..............................................................T..............C....................T.....................

2014KSU27.8 1321 .........................................T...........G........T..............C..........................................

2014KSU2091.1 1319 .......................G...........A....................G.....T.....A.....G..........................G.....G........G..T

2015KSU2327.2 1319 .......................G...........A....................G.....T.....A.....G..........................G.....G........----

2016KSU47.5 1321 .......................G.....G..A..A....................G.....T.....A.....G..........................G.....G........G..T

2014UMN3951.1 1319 .......................G.....G.....A....................G.....T.....A.....G.........................................----

2015UMN193.3 1321 .......................G.....G..A..A....................G.....T.....A..............................................-----

2015UMN370.6 1321 ..............................................................T..............C....................T.....................

2015UMN64.3 1321 ............A...........................................................................................................

2015UMN1735.7 1321 .......................G...........A....................G.....T.....A.....G..........................G.....G........----

2014UMN3624.74 1321 .......................G...........A....................G.....T.....A.....G..........................G.....G........G..T

2014UMN3720.5 1321 .......................G.....G.....A....................G.....T.....A.....G..........................G..............----

2014UMN3930.3 1321 .......................G...........A....................G.....T.....A.....G..........................G.....G........----

2014UMN3624.71 1321 .......................G...........A....................G.....T.....A.....G..........................G.....G........G..T

2015UMN865.5 1321 .......................G.....G.....A....................G.....T.....A.....G..........................G..................

2015UMN4053.5 1321 .......................G...........A....................G.....T.....A.....G..........................G.....G........G..T

05ZYH33 1441 GCATGACTATGTCTAAGACCTTTCCCTGTTATAACAGGCACTCCTACTTGCTGCGCTTTACGTTTAATGATACGACAAATGGTTGATTTACAAAAAGGATCTCCAAATCTTGAAATGATA

2016UMN1917.1 1441 .................................................................G......................................................

2016UMN1440.1 1437 -------------------------......................C.....A.....C.....G..A..G................................................

2014UMN3714.6 1441 .............................----------..............A.....C.....G..A..G.................G..............................

2016UMN146.4 1437 ---------------------------------------........C.....A.....C.....G..A..G.................G..............................

2016UMN668.5 1441 ...................-----.......................C.....A.....C.....G..A..G................................................

2016UMN1367.1,

2017UMN1574.421, and

2017KSU1528.8 1441 .....----------------------------------........C.....A.....C.....G..A..G.................G..............................

2016UMN2465.63B 1432 ------------------------.......................C.....A.....C.....G..A..G................................................

2016UMN2500.63 1440 ---------------------------------------........C.....A.....C.....G..A..G.................G..............................

2015UMN592.5 1441 .....-------------------.......................C.....A.....C.....G..A..G................................................

2016UMN1524.93 1412 ---------------------------------------........C..T..A.....C.....G.....G.................G..............................

2017UMN236.4 1427 ---------------------------------------........C..T..A.....C.....G.....G.................G..............................

2017UMN631.2 1441 .....G.......-----------------------...........C.....A.....C.....G..A..G.................G..............................

2016UMN2813.1 1441 .............................-------...........C.....A.....C.....G..A..G.................G..............................

2016UMN3027.6 1441 .....G.......--------------------------........C.....A.....C.....G..A..G.................G..............................

2014UMN2148.7 1437 ---------------------------------------........C.....A.....C.....G..A..G................................................

2017UMN55.9 1441 .....-------------------------------...........C.....A.....C.....G..A..G.................G..............................

2017UMN92.8 1441 .....-------------------.....A..T..T..A........C.....A.....C.....G..A..G................................................

2017UMN144.4 1441 .............--------------------------........C.....A.....C.....G..A..G.................G..............................

2017UMN145.1 1441 ..............-------------------------........C.....A.....C.....G..A..G................................................

2017UMN164.7 1441 .....----------------------------------........C.....A.....C.....G..A..G.................G..............................

2015UMN2795.3 and

2017UMN296.1 1441 .................................................................G......................................................

2017UMN1161.8 1441 .....G........G..............----------........C.....A.....C.....G..A..G.................G..............................

2015UMN1635.9 1441 .............................A....TT..T..........................G......................................................

2014KSU27.8 1441 .............................A....TT..T..............A...........G.............................T....T...................

2014KSU2091.1 1439 .....-------------------------------...........C.....A.....C.....G..A..G.................G..............................

2015KSU2327.2 1435 ------------------------------------------.....C.....A.....C.....G..A..G.................G..............................

2016KSU47.5 1441 .....G.............--------------------........C..T..A.....C.....G.....G.................G..............................

2014UMN3951.1 1435 ------------------------.......................C.....A.....C.....G..A..G................................................

2015UMN193.3 1436 ---------------------------------------........C..T..A.....C.....G.....G.................G..............................

2015UMN370.6 1441 .............................A....TT..T..........................G......................................................

2015UMN64.3 1441 .................................................................G..............A.......................................

2015UMN1735.7 1437 ---------------------------------------........C.....A.....C.....G..A..G.................G..............................

2014UMN3624.74 1441 .............................----------........C.....A.....C.....G..A..G.................G..............................

2014UMN3720.5 1437 ------------------------.....A..T..T..A........C.....A.....C.....G..A..G................................................

2014UMN3930.3 1437 ---------------------------------------..............A.....C.....G..A..G.................G..............................

2014UMN3624.71 1441 ..............-------------------------........C.....A.....C.....G..A..G.................G..............................

2015UMN865.5 1441 .....-------------------..............A........C.....A.....C.....G..A..G................................................

2015UMN4053.5 1441 .....----------------------------------........C.....A.....C.....G..A..G.................G..............................

05ZYH33 1561 AAATCTGTATCTTGATTTGCAAACTGATTTTTTCGCCAAACTTGAAGTACTTCAATAGTTATATCATCCAATTCAATAAGTCGTTCACCTGCTGGAGTCTTTGTTTGGTCTTTACGATAC

2016UMN1917.1 1561 ........C..................................................A............................................................

2016UMN1440.1 1532 ...........................................................A............................................................

2014UMN3714.6 1551 ...........................................................A......................................T.....................

2016UMN146.4 1518 ...........................................................A......................................T.....................

2016UMN668.5 1556 ........................................................................................................................

2016UMN1367.1,

2017UMN1574.421, and

2017KSU1528.8 1527 ...........................................................A......................................T.....................

2016UMN2465.63B 1528 ........................................................................................................................

2016UMN2500.63 1521 ...........................................................A......................................T.....................

2015UMN592.5 1542 ........................................................................................................................

2016UMN1524.93 1493 ...........................................................A............................................................

2017UMN236.4 1508 ...........................................................A............................................................

2017UMN631.2 1538 ...........................................................A......................................T.....................

2016UMN2813.1 1554 ...........................................................A......................................T.....................

2016UMN3027.6 1535 ...........................................................A......................................T.....................

2014UMN2148.7 1518 ...........................................................A............................................................

2017UMN55.9 1530 ...........................................................A......................................T.....................

2017UMN92.8 1542 ........................................................................................................................

2017UMN144.4 1535 ...........................................................A......................................T.....................

2017UMN145.1 1536 ...............................................G...........A......................................T.....................

2017UMN164.7 1527 ...........................................................A......................................T.....................

2015UMN2795.3 and

2017UMN296.1 1561 ........C..................................................A............................................................

2017UMN1161.8 1551 ...........................................................A......................................T.....................

2015UMN1635.9 1561 ...........................................................A............................................................

2014KSU27.8 1561 .............T........................................T....A.......................................................A....

2014KSU2091.1 1528 ...........................................................A......................................T.....................

2015KSU2327.2 1513 ...........................................................A......................................T.....................

2016KSU47.5 1541 .........G.................................................A............................................................

2014UMN3951.1 1531 ........................................................................................................................

2015UMN193.3 1517 .........G.................................................A............................................................

2015UMN370.6 1561 ...........................................................A............................................................

2015UMN64.3 1561 ...............................................C........................................................................

2015UMN1735.7 1518 ...........................................................A......................................T.....................

2014UMN3624.74 1551 ...........................................................A......................................T.....................

2014UMN3720.5 1533 ........................................................................................................................

2014UMN3930.3 1518 ...........................................................A......................................T.....................

2014UMN3624.71 1536 ...........................................................A......................................T.....................

2015UMN865.5 1542 ........................................................................................................................

2015UMN4053.5 1527 ...........................................................A......................................T.....................

05ZYH33 1681 CAATTTCCATTTTCATCTTTCTCCAAGGTTGTATGTACCTTTAGAAATTTTTTATCAAAATCAATATCCTCCCAACATAAAGATAAACCTTCACTCACTCGTACACCTGTCATATAATAT

2016UMN1917.1 1681 ..................................................A..T..............T...................................................

2016UMN1440.1 1652 ..................................................A..T..............T...................................................

2014UMN3714.6 1671 .....C.................T....................................................................G...........T...............

2016UMN146.4 1638 .....C.................T....................................................................G...........T...............

2016UMN668.5 1676 ..................................................A..T..................................................................

2016UMN1367.1,

2017UMN1574.421, and

2017KSU1528.8 1647 .....C.................T....................................................................G...........T...............

2016UMN2465.63B 1648 ..................................................A..T..................................................................

2016UMN2500.63 1641 .....C.................T....................................................................G...........T...............

2015UMN592.5 1662 ..................................................A..T..................................................................

2016UMN1524.93 1613 .....A.................T....................................................................G...........................

2017UMN236.4 1628 .....A.................T....................................................................G...........................

2017UMN631.2 1658 .....C.................T....................................................................G...........T...............

2016UMN2813.1 1674 .....C.................T....................................................................G...........T...............

2016UMN3027.6 1655 .....C.................T....................................................................G...........T...............

2014UMN2148.7 1638 ..................................................A..T..............T...................................................

2017UMN55.9 1650 .....C.................T....................................................................G...........T...............

2017UMN92.8 1662 ..................................................A..T..................................................................

2017UMN144.4 1655 .....C.................T....................................................................G...........T...............

2017UMN145.1 1656 .....C.................T....................................................................G...........................

2017UMN164.7 1647 .....C.................T....................................................................G...........T...............

2015UMN2795.3 and

2017UMN296.1 1681 ..................................................A..T..............T...................................................

2017UMN1161.8 1671 .....C.................T....................................................................G...........T...............

2015UMN1635.9 1681 .....C..............T..T........................................................C...........G...........................

2014KSU27.8 1681 .....C.................T....................................................................G...........................

2014KSU2091.1 1648 .....C.................T....................................................................G...........T...............

2015KSU2327.2 1633 .....C.................T....................................................................G...........T...............

2016KSU47.5 1661 .....A.................T....................................................................G...........................

2014UMN3951.1 1651 ..................................................A..T..................................................................

2015UMN193.3 1637 .....A.................T....................................................................G...........................

2015UMN370.6 1681 .....C..............T..T..........................A..T..............T...........C...........G...........................

2015UMN64.3 1681 ........................................................................................................................

2015UMN1735.7 1638 .....C.................T....................................................................G...........T...............

2014UMN3624.74 1671 .....C.................T....................................................................G...........T...............

2014UMN3720.5 1653 ..................................................A..T..................................................................

2014UMN3930.3 1638 .....C.................T....................................................................G...........T...............

2014UMN3624.71 1656 .....C.................T....................................................................G...........T...............

2015UMN865.5 1662 ..................................................A..T..................................................................

2015UMN4053.5 1647 .....C.................T....................................................................G...........T...............

05ZYH33 1801 AACCAGATGGCAGTAAATCGTTGTAATTCTTCATAATCATGTAAGTCAAATGATTTAATAAATGTCTGAAACTCCGCATAGGTCCAAAATGGAGTTTCTGGATGCTTACCTCTAGGATTA

2016UMN1917.1 1801 ........................................................G.C.............................................................

2016UMN1440.1 1772 ........................................................G.C.............................................................

2014UMN3714.6 1791 .G.....................C................................G...................A...........................................

2016UMN146.4 1758 .G.....................C................................G...................A...........................................

2016UMN668.5 1796 ...........T............................T...............G...............................................................

2016UMN1367.1,

2017UMN1574.421, and

2017KSU1528.8 1767 .G.....................C................................G...................A...........................................

2016UMN2465.63B 1768 ...........T............................T...............G...............................................................

2016UMN2500.63 1761 .G.....................C................................G...................A...........................................

2015UMN592.5 1782 ...........T............................T...............G...............................................................

2016UMN1524.93 1733 .......................C................................G.................................A.............T...............

2017UMN236.4 1748 .......................C................................G.................................A.............T...............

2017UMN631.2 1778 .G.....................C................................G...................A...........................................

2016UMN2813.1 1794 .G.....................C................................G...................A...........................................

2016UMN3027.6 1775 .G.....................C................................G...................A...........................................

2014UMN2148.7 1758 ........................................................G.C.............................................................

2017UMN55.9 1770 .G.....................C................................G...................A...........................................

2017UMN92.8 1782 ...........T............................T...............G...............................................................

2017UMN144.4 1775 .G.....................C................................G...................A...........................................

2017UMN145.1 1776 .......................C................................G...................T...........................................

2017UMN164.7 1767 .G.....................C................................G...................A...........................................

2015UMN2795.3 and

2017UMN296.1 1801 ........................................................G.C.............................................................

2017UMN1161.8 1791 .G.....................C................................G...................A...........................................

2015UMN1635.9 1801 .......................C................................G...............................................................

2014KSU27.8 1801 .......................C.................C..............G...............................................................

2014KSU2091.1 1768 .G.....................C................................G...................A...........................................

2015KSU2327.2 1753 .G.....................C................................G...................A...........................................

2016KSU47.5 1781 .......................C................................G...............................................T...............

2014UMN3951.1 1771 ...........T............................T...............G...............................................................

2015UMN193.3 1757 .......................C................................G...............................................T...............

2015UMN370.6 1801 ........................................................G...............................................................

2015UMN64.3 1801 ........................................................G........................................T......................

2015UMN1735.7 1758 .G.....................C................................G...................A...........................................

2014UMN3624.74 1791 .G.....................C................................G...................A...........................................

2014UMN3720.5 1773 ...........T............................T...............G...............................................................

2014UMN3930.3 1758 .G.....................C................................G...................A...........................................

2014UMN3624.71 1776 .G.....................C................................G...................A...........................................

2015UMN865.5 1782 ...........T............................T...............G...............................................................

2015UMN4053.5 1767 .G.....................C................................G...................A...........................................

05ZYH33 1921 TCTAATGCTTTACAAGGCATATCTGAAATATAGCCAAGTCTTTCTGCATAACCCATACAAGCTTTAAACCTTGACCATAAATTTTTCGCATAGTTTTCAGAATAATTTTCTATGATATGC

2016UMN1917.1 1921 .......................................................................................................................T

2016UMN1440.1 1892 ........................................................................................................................

2014UMN3714.6 1911 ......................T.................................................................................G..C............

2016UMN146.4 1878 ......................T.................................................................................G..C............

2016UMN668.5 1916 ........................................................................................................................

2016UMN1367.1,

2017UMN1574.421, and

2017KSU1528.8 1887 ......................T.................................................................................G..C............

2016UMN2465.63B 1888 ........................................................................................................................

2016UMN2500.63 1881 ......................T.................................................................................G..C............

2015UMN592.5 1902 ........................................................................................................................

2016UMN1524.93 1853 ......................T....................................................................................C............

2017UMN236.4 1868 ......................T....................................................................................C............

2017UMN631.2 1898 ......................T.................................................................................G..C............

2016UMN2813.1 1914 ......................T.................................................................................G..C............

2016UMN3027.6 1895 ......................T.................................................................................G..C............

2014UMN2148.7 1878 ........................................................................................................................

2017UMN55.9 1890 ......................T.................................................................................G..C............

2017UMN92.8 1902 ........................................................................................................................

2017UMN144.4 1895 ......................T.................................................................................G..C............

2017UMN145.1 1896 ......................T.................................................................................G..C............

2017UMN164.7 1887 ......................T.................................................................................G..C............

2015UMN2795.3 and

2017UMN296.1 1921 .......................................................................................................................T

2017UMN1161.8 1911 ......................T.................................................................................G..C............

2015UMN1635.9 1921 ......................T.................................................................................G..C............

2014KSU27.8 1921 ......................T.................................................................................G..C............

2014KSU2091.1 1888 ......................T.................................................................................G..C............

2015KSU2327.2 1873 ......................T.................................................................................G..C............

2016KSU47.5 1901 ......................T....................................................................................C............

2014UMN3951.1 1891 ........................................................................................................................

2015UMN193.3 1877 ......................T....................................................................................C............

2015UMN370.6 1921 .......................................................................................................................T

2015UMN64.3 1921 ........................................................................................................................

2015UMN1735.7 1878 ......................T.................................................................................G..C............

2014UMN3624.74 1911 ......................T.................................................................................G..C............

2014UMN3720.5 1893 ........................................................................................................................

2014UMN3930.3 1878 ......................T.................................................................................G..C............

2014UMN3624.71 1896 ......................T.................................................................................G..C............

2015UMN865.5 1902 ........................................................................................................................

2015UMN4053.5 1887 ......................T.................................................................................G..C............

05ZYH33 2041 AATCTAAAAGCCTCGCAATCT

2016UMN1917.1 2041 .....................

2016UMN1440.1 2012 .....................

2014UMN3714.6 2031 ...........T.........

2016UMN146.4 1998 ...........T.........

2016UMN668.5 2036 ...........T.........

2016UMN1367.1,

2017UMN1574.421, and

2017KSU1528.8 2007 ...........T.........

2016UMN2465.63B 2008 ...........T.........

2016UMN2500.63 2001 ...........T.........

2015UMN592.5 2022 ...........T.........

2016UMN1524.93 1973 ...........T.........

2017UMN236.4 1988 ...........T.........

2017UMN631.2 2018 ...........T.........

2016UMN2813.1 2034 ...........T.........

2016UMN3027.6 2015 ...........T.........

2014UMN2148.7 1998 .....................

2017UMN55.9 2010 ...........T.........

2017UMN92.8 2022 ...........T.........

2017UMN144.4 2015 ...........T.........

2017UMN145.1 2016 ...........T.........

2017UMN164.7 2007 ...........T.........

2015UMN2795.3 and

2017UMN296.1 2041 .....................

2017UMN1161.8 2031 ...........T.........

2015UMN1635.9 2041 ...........T.........

2014KSU27.8 2041 ...........T.........

2014KSU2091.1 2008 ...........T.........

2015KSU2327.2 1993 ...........T.........

2016KSU47.5 2021 ...........T.........

2014UMN3951.1 2011 ...........T.........

2015UMN193.3 1997 ...........T.........

2015UMN370.6 2041 .....................

2015UMN64.3 2041 .....................

2015UMN1735.7 1998 ...........T.........

2014UMN3624.74 2031 ...........T.........

2014UMN3720.5 2013 ...........T.........

2014UMN3930.3 1998 ...........T.........

2014UMN3624.71 2016 ...........T.........

2015UMN865.5 2022 ...........T.........

2015UMN4053.5 2007 ...........T.........

# **89K PAI CH5/CH6**

05ZYH33 1 ATAAATAGCCCCATCCTCATCAATCCAACCTTTACTCAAAGACAACTCCAACCGATCTTTTAAAACCGCGTAGGCTACCTTAACCTCCAGCTTCATATCCTTATAACGTTCACTCTCAAA

2016UMN1917.1 1 ...........................................................................................................A............

2014UMN2148.7,

2015UMN3436.5,

2014UMN1785.4,

2015UMN56.5, and

2016UMN1470.8 1 ........................................................................................................................

2016UMN1440.1 1 .........................................................Y..........................Y..............Y....................

2016UMN917.8 and

2014UMN3704.91B 1 ...........................G............................................................................................

2014UMN3624.74 and

2014UMN3714.6 1 .........................................................C..........................T..............Y....................

2016UMN102.5 1 ...........................G............................................................................................

2016UMN146.4 1 ...........................G..C...........T..............C..............................................................

2016UMN209.8 1 .........................................................C..........................T..............T....................

2014UMN3786.4 1 .........................................................C..........................T..............T....................

2016UMN668.5 1 .........................................................C..........................T...................................

2014UMN3368.9 and

2016UMN924.3 1 ...........................R.............................Y..........................Y..............Y....................

2016UMN1367.1 and

2017UMN144.4 1 ...........................G..C...........T..............C..............................................................

2015UMN3869.3 1 ...........................R.............................Y..........................Y..............Y....................

2015UMN592.5,

2014UMN3720.5,

2015UMN865.5, and

2016UMN2465.63B 1 .........................................................C..........................T...................................

2016UMN2500.63 1 .................T.........G..C...........T..............C..............................................................

2016UMN2500.62B 1 .........................................................C..........................T..............T....................

2014UMN3928.6 1 ...........................G............................................................................................

2015UMN27.5 1 ...........................G............................................................................................

2015UMN122.1 1 ...........................G............................................................................................

2017UMN355.91 1 .........................................................C..........................T..............T....................

2016UMN1524.93 1 ......T....................G..............................................................T..............C..............

2017UMN236.4 1 ...........................G..............................................................T..............C..............

2014UMN3624.71,

2017UMN631.2,

2016UMN2813.1,

2016UMN3027.6,

2017UMN55.9,

2017UMN164.7,

2017UMN1574.421, and

2014KSU2091.1 1 ...........................G..C...........T..............C..............................................................

2015UMN1502.6,

2015UMN1502.7,

2016UMN2677.3, and

2017UMN539.9 1 ............................................R........K...Y..........................T..............Y....................

2016UMN2940.9 1 ...........................G............................................................................................

2016UMN3248.4 1 ...........................G............................................................................................

2016UMN3250.3 1 ...........................G............................................................................................

2017UMN145.1 1 ...T.....................................................C..........................T..............T....................

2017UMN296.1 1 ...........................R.............................Y..........................Y..............Y....................

2017UMN834.1 1 ............................................G.......................................T...................................

2017UMN991.6 1 .........................................................C..........................T..............T....................

2017UMN1049.1 1 ...........................R.............................Y..........................Y..............Y....................

2017UMN1161.8 1 ...........................R..Y...........Y..............C..........................Y..............Y....................

2015UMN1635.9 1 -..........................G..C..........................................................................C..............

2017KSU1528.8 1 ...........................R..Y...........Y..............C..........................Y..............Y....................

2015UMN2473.3 1 ...........................G............................................................................................

2014KSU27.8 1 -..........................G..C.............G........T..............................T...................................

2015UMN2626.7 1 .........................................................C..........................T..............T....................

2015KSU2327.2 1 ...........................G..C...........T..............C..............................................................

2016KSU47.5 1 ...........................R..Y..........................Y..........................Y..............Y.....M..............

2015UMN2795.3 1 ...........................R.............................Y..........................Y..............Y....................

2015UMN2869.3 1 .........................................................C..........................T..............T....................

2015UMN3222.1 1 ...........................R.............................Y..........................Y..............Y....................

2014UMN3951.1 1 -..............T...........G..C..........................................................................C..............

2015UMN43.3 1 ...........................G............................................................................................

2015UMN193.3 1 -..........................G..C..........................................................................C..............

2015UMN367.3 1 ............................................G.......................................T....................C..............

2015UMN370.6 1 -..........................G..C..........................C..........................T...................................

2015UMN1919.5 1 .........................................................C..........................T..............T....................

2014UMN3749.9 1 ...........................G............................................................................................

2015UMN64.3 1 ............................................G........T...................................A..............................

2015UMN277.7 1 .........................................................C..........................T..............T....................

2015UMN1735.7 1 .....................................................T..................................................................

2014UMN3631.1 1 .........................................................C..........................T..............T....................

2014UMN3930.3 1 .........................................................C..........................Y..............Y....................

2015UMN4145.9 1 ...........................G............................................................................................

2015UMN4053.5 1 ...........................R..Y..........................C..........................T..............Y.....M..............

05ZYH33 121 CAAAATTTTAGGGAGTTTATAATAACGCTCTGATGTTTGATATTGATTAGCGGTAATACGTTTCATGATTGTCCCTCCAAGACTAATATTCCAACATTTCCAAATTCATCAAATCGGATT

2016UMN1917.1 121 ..................G.....................................................................................................

2014UMN2148.7,

2015UMN3436.5,

2014UMN1785.4,

2015UMN56.5, and

2016UMN1470.8 121 ...G.C...T..........................C.....C...........................A...............A.G............G..................

2016UMN1440.1 121 ...G.C...T..........................C............................C......................................................

2016UMN917.8 and

2014UMN3704.91B 121 ...G.C...T..........................C....................T............................A.................................

2014UMN3624.74 and

2014UMN3714.6 121 ...G.C...T..........................C.....Y..............W.......Y....................W.K............R..................

2016UMN102.5 121 ...G.C...T..........................C....................T............................A.................................

2016UMN146.4 121 ..........................................C..............T............................A.G.....A...A..G..................

2016UMN209.8 121 ...G.C...T..........................C............................C..............................................--------

2014UMN3786.4 121 ...G.C...T..........................C............................C......................................................

2016UMN668.5 121 ...G.C...T..........................C.................................A...............A.G............G..................

2014UMN3368.9 and

2016UMN924.3 121 ...G.C...T..........................C....................W.......Y....................W.................................

2016UMN1367.1 and

2017UMN144.4 121 ..........................................C..............T............................A.G.....A...A..G..................

2015UMN3869.3 121 ...G.C...T..........................C....................W.......Y....................W.................................

2015UMN592.5,

2014UMN3720.5,

2015UMN865.5, and

2016UMN2465.63B 121 ...G.C...T..........................C.................................A...............A.G............G..................

2016UMN2500.63 121 ..........................................C..............T............................A.G.....A...A..G..................

2016UMN2500.62B 121 ...G.C...T..........................C.................................A...............A.G............G..................

2014UMN3928.6 121 ...G.C...T..........................C....................T............................A.................................

2015UMN27.5 121 ...G.C...T..........................C....................T............................A.................................

2015UMN122.1 121 ...G.C...T..........................C....................T............................A.................................

2017UMN355.91 121 ...G.C...T..........................C............................C..............................................--------

2016UMN1524.93 121 .....C..................T...........C.....C.............................................................................

2017UMN236.4 121 .....C..................T...........C.....C.............................................................................

2014UMN3624.71,

2017UMN631.2,

2016UMN2813.1,

2016UMN3027.6,

2017UMN55.9,

2017UMN164.7,

2017UMN1574.421, and

2014KSU2091.1 121 ..........................................C..............T............................A.G.....A...A..G..................

2015UMN1502.6,

2015UMN1502.7,

2016UMN2677.3, and

2017UMN539.9 121 ...G.C...T..........................C............................Y....................W.................................

2016UMN2940.9 121 ...G.C...T..........................C....................T............................A.................................

2016UMN3248.4 121 ...G.C...T..........................C....................T............................A.................................

2016UMN3250.3 121 ...G.C...T..........................C....................T............................A.................................

2017UMN145.1 121 ...G.C...T..........................C............................C..............................................--------

2017UMN296.1 121 ...G.C...T..........................C....................W.......Y....................W.................................

2017UMN834.1 121 ...G.C...T..........................C.................................A...............A.G............G..................

2017UMN991.6 121 ...G.C...T..........................C............................C..............................................--------

2017UMN1049.1 121 ...G.C...T..........................C....................W.......Y....................W.................................

2017UMN1161.8 121 ...R.Y...W..........................Y.....Y..............W............R...............A.G.....M...W..G..................

2015UMN1635.9 120 .....C...G..........................C..............T.....T............................A.................................

2017KSU1528.8 121 ...R.Y...W..........................Y.....Y..............W.......Y......................................................

2015UMN2473.3 121 ...G.C...T..........................C....................T............................A.................................

2014KSU27.8 120 ...G.C...T..........................C.................................A...............A.G............G..................

2015UMN2626.7 121 ...G.C...T..........................C............................C..............................................--------

2015KSU2327.2 121 ..........................................C..............T............................A.G.....A...A..G..................

2016KSU47.5 121 ...R.C...G..........................C..............K.....T............................A.................................

2015UMN2795.3 121 ...G.C...T..........................C....................W.......Y....................W.................................

2015UMN2869.3 121 ...G.C...T..........................C............................C..............................................--------

2015UMN3222.1 121 ...G.C...T..........................C....................W.......Y....................W.................................

2014UMN3951.1 120 .....C............G.................C.................................................G...................T...C.........

2015UMN43.3 121 ...G.C...T..........................C....................T............................A.................................

2015UMN193.3 120 .....C...G..........................C..............T.....T............................A.................................

2015UMN367.3 121 ...G.C...T..........................C.................................A...............A.G............G..................

2015UMN370.6 120 ...G.C...T..........................C.................................A...............A.G............G..................

2015UMN1919.5 121 ...G.C...T..........................C............................Y....R..............................R..................

2014UMN3749.9 121 ...G.C...T..........................C....................T............................A.................................

2015UMN64.3 121 ...G.C...T...............................................T............................A.................................

2015UMN277.7 121 ...G.C...T..........................C............................C..............................................--------

2015UMN1735.7 121 ....................................C.....C..............T......................A.....A.................................

2014UMN3631.1 121 ...G.C...T..........................C............................C..............................................--------

2014UMN3930.3 121 ...R.Y...W..........................C............................C......................................................

2015UMN4145.9 121 ...G.C...T..........................C....................T............................A.................................

2015UMN4053.5 121 ...G.C...T..........................C.....Y..............W.......Y....................W.K............R..................

05ZYH33 241 AAACCTAGTTGTTCCATTTCATCAATTAGCTGTGTTGCTTTCTCAATATCAACTCCTAAAATAGCCACGAGATGACACATCACGATTTGGCGTGATGATTGTTCCTTCTTTTGCATAATT

2016UMN1917.1 241 ............A...................A..........................G....T.......................................AAA.........G...

2014UMN2148.7,

2015UMN3436.5,

2014UMN1785.4,

2015UMN56.5, and

2016UMN1470.8 241 .......C........A........CC..A..A.........................................G.........................T...............G...

2016UMN1440.1 241 .......C........A........CC..A..A.........................................R..-......................T...............G...

2016UMN917.8 and

2014UMN3704.91B 241 .......C........A........CC..A..A.......................................................................................

2014UMN3624.74 and

2014UMN3714.6 241 ................................A...............................................................................T...G...

2016UMN102.5 241 .......C........A........CC..A..A.......................................................................................

2016UMN146.4 241 .......................-----------------------......................A.....G..............A................C.............

2016UMN209.8 233 -----------------...............A....T....A.....................T............T..................................T.......

2014UMN3786.4 241 ................................A....T....A.....................T............T..................................T.......

2016UMN668.5 241 .......C........A........CC..A..A...........T.............................G.............................................

2014UMN3368.9 and

2016UMN924.3 241 .......C........A........CC..A..A.......................................................................................

2016UMN1367.1 and

2017UMN144.4 241 .......C........A...................................................A.....G..............A................C.............

2015UMN3869.3 241 .......C........A........CC..A..A...............................................................................K.......

2015UMN592.5,

2014UMN3720.5,

2015UMN865.5, and

2016UMN2465.63B 241 .......C........A........CC..A..A...........T.............................G.............................................

2016UMN2500.63 241 .......C........A........CC..A..A...................................A.....G..............A................C.............

2016UMN2500.62B 241 .......C........A........C...A..A.......................----............................................T.G.....T.......

2014UMN3928.6 241 .......C........A........CC..A..A.......................................................................................

2015UMN27.5 241 .......C........A........CC..A..A.......................................................................................

2015UMN122.1 241 .......C........A........CC..A..A.......................................................................................

2017UMN355.91 233 -----------------...............A....T....A.....................T............T..................................T.......

2016UMN1524.93 241 .......C........................A.......................C..G....A.G.....................................................

2017UMN236.4 241 .......C........................A.......................C..G....A.G.....................................................

2014UMN3624.71,

2017UMN631.2,

2016UMN2813.1,

2016UMN3027.6,

2017UMN55.9,

2017UMN164.7,

2017UMN1574.421, and

2014KSU2091.1 241 .......C........A........CC..A..A...................................A.....G..............A................C.............

2015UMN1502.6,

2015UMN1502.7,

2016UMN2677.3, and

2017UMN539.9 241 .......C........................A..............T........Y..R....T.........R..Y..M.......................................

2016UMN2940.9 241 .......C........A........CC..A..A.......................................................................................

2016UMN3248.4 241 .......C........A........CC..A..A.......................................................................................

2016UMN3250.3 241 .......C........A........CC..A..A.......................................................................................

2017UMN145.1 233 -----------------...............A....T....A.....................T............T..................................T.......

2017UMN296.1 241 .......C..................C..A..A..................................................C.....A..............................

2017UMN834.1 241 .......C........A........CA..A..A.........................................G.......................C.....A...........G...

2017UMN991.6 233 -----------------...............A....T....A.....................T............T..................................T.......

2017UMN1049.1 241 .......C........A........CC..A..A...............................Y............Y..................................K.......

2017UMN1161.8 241 .......C........A........C...A..A...................................A.....G..............A................C.............

2015UMN1635.9 240 .......C........................A.......................C..G.......................C.....A................C.............

2017KSU1528.8 241 .......C........A...............A....-....W.....................Y...R.....R..Y...........R................Y.....K.......

2015UMN2473.3 241 .......C........A........CC..A..A.......................................................................................

2014KSU27.8 240 .......C........................AA........A................G....T............T..........................................

2015UMN2626.7 233 -----------------...............A....T....A.....................T............T..................................T.......

2015KSU2327.2 241 .......C........A........CC..A..A...................................A.....G..............A................C.............

2016KSU47.5 241 .......C........................A.......................C..G.......................C.....A................C.............

2015UMN2795.3 241 .......C.................CC..A..A...............................Y............Y.....S.....R..............................

2015UMN2869.3 233 -----------------...............A....T....A.....................T............T..................................T.......

2015UMN3222.1 241 .......C........A........CC..A..A...............................Y............Y..................................K.......

2014UMN3951.1 240 .........................C......A.......................C..G....T.........G..............A..............................

2015UMN43.3 241 .......C........A........CC..A..A.......................................................................................

2015UMN193.3 240 .......C........................A.......................C..G.......................C.....A................C.............

2015UMN367.3 241 .......C........A........CC..A..A...................................................................................G...

2015UMN370.6 240 .......C........A...........................T.............................G........T............A.......................

2015UMN1919.5 241 .......C........A........C...M..A....Y....W.....................T............Y..........................Y.K.....T.......

2014UMN3749.9 241 .......C........A........CC..A..A.......................................................................................

2015UMN64.3 241 .......C........A........CC..A..A...............................Y............Y..................................K.......

2015UMN277.7 233 -----------------...............A....T....A.....................T............T..................................T.......

2015UMN1735.7 241 .......C........A........CC..A..A...............................T............T......................................G...

2014UMN3631.1 233 ---------------------...........A....T....A.....................T............T..................................T.......

2014UMN3930.3 241 .......C........A...................................................A.....G..............A................C.............

2015UMN4145.9 241 .......C........A........CC..A..A.......................................................................................

2015UMN4053.5 241 ................................A...............................................................................T...G...

05ZYH33 361 ACCTCCAAAACGCAAAAAAAGGGGCAGACAATTTAATGTCTACCCCGAAAATTTATTAAAACAAAAATCCTGCCAAAGAATTTTTGGCAGGATTTTTGGCAGGAAACCAAATCAATTTAT

2016UMN1917.1 361 ........................T.......CA..................A........................T.................C........................

2014UMN2148.7,

2015UMN3436.5,

2014UMN1785.4,

2015UMN56.5, and

2016UMN1470.8 361 ........................T...............................................................................................

2016UMN1440.1 360 ........................T...............................................................................................

2016UMN917.8 and

2014UMN3704.91B 361 ......----------------------------------------------------..............................................................

2014UMN3624.74 and

2014UMN3714.6 361 ........................T...............................................................................................

2016UMN102.5 361 .........------------------------------------------------...............................................................

2016UMN146.4 338 ........................T...........................A...................................................................

2016UMN209.8 336 ......---------------------------------------------------...............................................................

2014UMN3786.4 361 ........................T...............................................................................................

2016UMN668.5 361 ........................T...............................................................................................

2014UMN3368.9 and

2016UMN924.3 361 ......-----------------------------------------------...................................................................

2016UMN1367.1 and

2017UMN144.4 361 ........................T...........................A...................................................................

2015UMN3869.3 361 ....................---------------------------------..........................T...................................A....

2015UMN592.5,

2014UMN3720.5,

2015UMN865.5, and

2016UMN2465.63B 361 ........................T...............................................................................................

2016UMN2500.63 361 ........................T...........................A...................................................................

2016UMN2500.62B 357 ......-----------------------------------------------..........................T...................................A....

2014UMN3928.6 361 ......-----------------------------------------------...................................................................

2015UMN27.5 361 ......---------------------------------------------------...............................................................

2015UMN122.1 361 ..........------------------------......................................................................................

2017UMN355.91 336 T.....-----------------------------------------------..........................T...................................A....

2016UMN1524.93 361 ........................T.......................T...............................A.......................................

2017UMN236.4 361 ........................T.......................T...............................A.......................................

2014UMN3624.71,

2017UMN631.2,

2016UMN2813.1,

2016UMN3027.6,

2017UMN55.9,

2017UMN164.7,

2017UMN1574.421, and

2014KSU2091.1 361 ........................T...........................A...................................................................

2015UMN1502.6,

2015UMN1502.7,

2016UMN2677.3, and

2017UMN539.9 361 ........................T...............................................................................................

2016UMN2940.9 361 ......-----------------------------------------------------------------.................................................

2016UMN3248.4 361 ......---------------------------------------------------......................T...................................A....

2016UMN3250.3 361 ......----------------------------------------------------.....................T...................................A....

2017UMN145.1 336 ......-----------------------------------------------..............................................................A....

2017UMN296.1 361 .......C................T.........................G.....................................................................

2017UMN834.1 361 .------------------------------.........................................................................................

2017UMN991.6 336 ......-----------------------------------------------...................................................................

2017UMN1049.1 361 ....................---------------------------------...................................................................

2017UMN1161.8 361 ........................T...........................A...................................................................

2015UMN1635.9 360 ...........A............T...............................................................................................

2017KSU1528.8 360 ........................T...........................A...................................................................

2015UMN2473.3 361 ......-----------------------------------------------..........................T...................................A....

2014KSU27.8 360 ........................T...............................................................................................

2015UMN2626.7 336 ......----------------------------------------------------------------------............................................

2015KSU2327.2 361 ........................T...........................A...................................................................

2016KSU47.5 361 ...........A............T...............................................................................................

2015UMN2795.3 361 .......C................T.........................G.....................................................................

2015UMN2869.3 336 ......-----------------------------------------------...................................................................

2015UMN3222.1 361 ......---------------------------------------------------......................T...................................A....

2014UMN3951.1 360 ...........A............G...............................................................................................

2015UMN43.3 361 ......------------------------------------------------------------------................................................

2015UMN193.3 360 ...........A............T...............................................................................................

2015UMN367.3 361 ........................T.......CA..................A..........................................C........................

2015UMN370.6 360 ........................T...............................................................................................

2015UMN1919.5 361 ......-----------------------------------------------...................................................................

2014UMN3749.9 361 ......----------------------------------------------------.....................T...................................A....

2015UMN64.3 361 ........................T.......C..G..............G.....................................................................

2015UMN277.7 336 ......-----------------------------------------------...................................................................

2015UMN1735.7 361 ........................TG......CA.............................................................C........................

2014UMN3631.1 332 ......---------------------------------------------------...............................................................

2014UMN3930.3 361 ........................T...........................A...................................................................

2015UMN4145.9 361 ......----------------------------------------------------..............................................................

2015UMN4053.5 361 ........................T...............................................................................................

05ZYH33 481 CAGTTTCTATCAATCGCTTATCGCTCTCAAAGACTGGTAAATAGGGATTCCGCAATCTAATTATGATGTGACTCTTATTTAAGAGTAACTGAAGCGCCTGCTTCTTCCAATTTAGCTTTG

2016UMN1917.1 481 ...............A........................................................................................................

2014UMN2148.7,

2015UMN3436.5,

2014UMN1785.4,

2015UMN56.5, and

2016UMN1470.8 481 ........................................................................................................................

2016UMN1440.1 480 ........................................................................................................................

2016UMN917.8 and

2014UMN3704.91B 429 ..........................................C...C......TC..A....GCT.....CT................................................

2014UMN3624.74 and

2014UMN3714.6 481 ........................................................................................................................

2016UMN102.5 433 ..........................................C...C......TC..A....GCT.....CT................................................

2016UMN146.4 458 ..................................................................................T.....................................

2016UMN209.8 405 ..........................................C...C......TC..A....GCT.....CT................................................

2014UMN3786.4 481 .................-----------..............C...C......TC..A....GCT.....CT................................................

2016UMN668.5 481 ........................................................................................................................

2014UMN3368.9 and

2016UMN924.3 434 ..........................................C...C......TC..A....GCT.....CT................................................

2016UMN1367.1 and

2017UMN144.4 481 ........................................................................................................................

2015UMN3869.3 448 ........T.A................A..............C...C......TC..A....GCT.....CT................................................

2015UMN592.5,

2014UMN3720.5,

2015UMN865.5, and

2016UMN2465.63B 481 ........................................................................................................................

2016UMN2500.63 481 ........................................................................................................................

2016UMN2500.62B 430 ........T.A.......................-------------------------------.......................................................

2014UMN3928.6 434 ..................----------..............C...C......TC..A....GCT.....CT................................................

2015UMN27.5 430 ..................----------..............C...C......TC..A....GCT.....CT................................................

2015UMN122.1 457 ..........................................C...C......TC..A....GCT.....CT................................................

2017UMN355.91 409 ..........................................C...C......TC..A....GCT.....CT................................................

2016UMN1524.93 481 ........................................................................................................................

2017UMN236.4 481 ........................................................................................................................

2014UMN3624.71,

2017UMN631.2,

2016UMN2813.1,

2016UMN3027.6,

2017UMN55.9,

2017UMN164.7,

2017UMN1574.421, and

2014KSU2091.1 481 ........................................................................................................................

2015UMN1502.6,

2015UMN1502.7,

2016UMN2677.3, and

2017UMN539.9 481 ........................................................................................................................

2016UMN2940.9 416 ..................----------..............C...C......TC..A....GCT.....CT................................................

2016UMN3248.4 430 ........T.A...............................C...C......TC..A....GCT.....CT................................................

2016UMN3250.3 429 ........T.A...............................C...C......TC..A....GCT.....CT................................................

2017UMN145.1 409 ........T.A...............................C...C......TC..A....GCT.....CT................................................

2017UMN296.1 481 ........................................................................................................................

2017UMN834.1 451 ...............................................................-----------..............................................

2017UMN991.6 409 ........---------------...................C...C......TC..A....GCT.....CT................................................

2017UMN1049.1 448 ...............-------------..............C...C......TC..A....GCT.....CT................................................

2017UMN1161.8 481 ........................................................................................................................

2015UMN1635.9 480 ........................................................................................................................

2017KSU1528.8 480 ........................................................................................................................

2015UMN2473.3 434 ........T.A...............................C...C......TC..A....GCT.....CT................................................

2014KSU27.8 480 ........................................................................................................................

2015UMN2626.7 386 ..........................................C...C......TC..A....GCT.....CT................................................

2015KSU2327.2 481 ........................................................................................................................

2016KSU47.5 481 ........................................................................................................................

2015UMN2795.3 481 ........................................................................................................................

2015UMN2869.3 409 ........................................................................................................................

2015UMN3222.1 430 ........T.A................A..............C...C......TC..A....GCT.....CT................................................

2014UMN3951.1 480 ........................................................................................................................

2015UMN43.3 415 ..................----------..............C...C......TC..A....GCT.....CT................................................

2015UMN193.3 480 ........................................................................................................................

2015UMN367.3 481 ........................................................................................................................

2015UMN370.6 480 ........................................................................................................................

2015UMN1919.5 434 ..........................................C...C......TC..A....GCT.....CT................................................

2014UMN3749.9 429 ........T.A................A..............C...C......TC..A....GCT.....CT................................................

2015UMN64.3 481 ........................................................................................................................

2015UMN277.7 409 ..........................................C...C......TC..A....GCT.....CT................................................

2015UMN1735.7 481 ...............................................................................................T..A........A.G..........

2014UMN3631.1 401 ..........................................C...C......TC..A....GCT.....CT................................................

2014UMN3930.3 481 ........................................................................................................................

2015UMN4145.9 429 ..................----------..............C...C......TC..A....GCT.....CT................................................

2015UMN4053.5 481 ........................................................................................................................

05ZYH33 601 ATTTCTTCAGCTTCTGCAGTTGCAACGCCTTCTTTAACCATAGCTGGTGCACCGTCAACAAGCTCTTTAGCTTCTTTAAGACCAAGACCAGTGATTTCACGTACAACTTTGATAACGCCA 2016UMN1917.1 601 ........................................................................................................................

2014UMN2148.7,

2015UMN3436.5,

2014UMN1785.4,

2015UMN56.5, and

2016UMN1470.8 601 ........................................................................................................................

2016UMN1440.1 600 ........................................................................................................................

2016UMN917.8 and

2014UMN3704.91B 549 ........................................................................................................................

2014UMN3624.74 and

2014UMN3714.6 601 ........................................................................................................................

2016UMN102.5 553 ........................................................................................................................

2016UMN146.4 578 ........................................................................................................................

2016UMN209.8 525 ........................................................................................................................

2014UMN3786.4 590 ........................................................................................................................

2016UMN668.5 601 ........................................................................................................................

2014UMN3368.9 and

2016UMN924.3 554 ........................................................................................................................

2016UMN1367.1 and

2017UMN144.4 601 ........................................................................................................................

2015UMN3869.3 568 ........................................................................................................................

2015UMN592.5,

2014UMN3720.5,

2015UMN865.5, and

2016UMN2465.63B 601 ........................................................................................................................

2016UMN2500.63 601 ........................................................................................................................

2016UMN2500.62B 519 ...........................T............................................................................................

2014UMN3928.6 544 ........................................................................................................................

2015UMN27.5 540 ........................................................................................................................

2015UMN122.1 577 ........................................................................................................................

2017UMN355.91 529 ........................................................................................................................

2016UMN1524.93 601 ........................................................................................................................

2017UMN236.4 601 ........................................................................................................................

2014UMN3624.71,

2017UMN631.2,

2016UMN2813.1,

2016UMN3027.6,

2017UMN55.9,

2017UMN164.7,

2017UMN1574.421, and

2014KSU2091.1 601 ........................................................................................................................

2015UMN1502.6,

2015UMN1502.7,

2016UMN2677.3, and

2017UMN539.9 601 ........................................................................................................................

2016UMN2940.9 526 ........................................................................................................................

2016UMN3248.4 550 ........................................................................................................................

2016UMN3250.3 549 ........................................................................................................................

2017UMN145.1 529 ........................................................................................................................

2017UMN296.1 601 ........................................................................................................................

2017UMN834.1 560 ........................................................................................................................

2017UMN991.6 514 ........................................................................................................................

2017UMN1049.1 555 ........................................................................................................................

2017UMN1161.8 601 ........................................................................................................................

2015UMN1635.9 600 ........................................................................................................................

2017KSU1528.8 600 ........................................................................................................................

2015UMN2473.3 554 ........................................................................................................................

2014KSU27.8 600 ........................................................................................................................

2015UMN2626.7 506 ........................................................................................................................

2015KSU2327.2 601 ..................................................G...........T.........................................................

2016KSU47.5 601 ........................................................................................................................

2015UMN2795.3 601 ........................................................................................................................

2015UMN2869.3 529 ........................................................................................................................

2015UMN3222.1 550 ........................................................................................................................

2014UMN3951.1 600 ........................................................................................................................

2015UMN43.3 525 ........................................................................................................................

2015UMN193.3 600 ........................................................................................................................

2015UMN367.3 601 ........................................................................................................................

2015UMN370.6 600 ........................................................................................................................

2015UMN1919.5 554 ........................................................................................................................

2014UMN3749.9 549 ........................................................................................................................

2015UMN64.3 601 ........................................................................................................................

2015UMN277.7 529 ........................................................................................................................

2015UMN1735.7 601 ..................................................G...........T...................................G..A..................

2014UMN3631.1 521 ........................................................................................................................

2014UMN3930.3 601 ........................................................................................................................

2015UMN4145.9 539 ........................................................................................................................

2015UMN4053.5 601 ........................................................................................................................

05ZYH33 721 ACTTTTTTGTCGCCAGCAGATGTCAATTCAACGTCGAATGAATCTTTAGCTTCTTCAGCAGCACCACCAGCTGCAGCAACAGCTACAGGAGCAGCCGCAGTTACACCAAATTCTTCTTCG

2016UMN1917.1 721 .........................................G..............................................................................

2014UMN2148.7,

2015UMN3436.5,

2014UMN1785.4,

2015UMN56.5, and

2016UMN1470.8 721 ........................................................................................................................

2016UMN1440.1 720 ........................................................................................................................

2016UMN917.8 and

2014UMN3704.91B 669 ........................................................................................................................

2014UMN3624.74 and

2014UMN3714.6 721 .........................................G........C.....................................................................

2016UMN102.5 673 ........................................................................................................................

2016UMN146.4 698 ........................................................................................................................

2016UMN209.8 645 ........................................................................................................................

2014UMN3786.4 710 ........................................................................................................................

2016UMN668.5 721 ........................................................................................................................

2014UMN3368.9 and

2016UMN924.3 674 ........................................................................................................................

2016UMN1367.1 and

2017UMN144.4 721 ........................................................................................................................

2015UMN3869.3 688 ........................................................................................................................

2015UMN592.5,

2014UMN3720.5,

2015UMN865.5, and

2016UMN2465.63B 721 ........................................................................................................................

2016UMN2500.63 721 ........................................................................................................................

2016UMN2500.62B 639 ........................................................................................................................

2014UMN3928.6 664 ........................................................................................................................

2015UMN27.5 660 ........................................................................................................................

2015UMN122.1 697 ........................................................................................................................

2017UMN355.91 649 ........................................................................................................................

2016UMN1524.93 721 ........................................................................................................................

2017UMN236.4 721 ........................................................................................................................

2014UMN3624.71,

2017UMN631.2,

2016UMN2813.1,

2016UMN3027.6,

2017UMN55.9,

2017UMN164.7,

2017UMN1574.421, and

2014KSU2091.1 721 ........................................................................................................................

2015UMN1502.6,

2015UMN1502.7,

2016UMN2677.3, and

2017UMN539.9 721 ........................................................................................................................

2016UMN2940.9 646 ........................................................................................................................

2016UMN3248.4 670 ........................................................................................................................

2016UMN3250.3 669 ........................................................................................................................

2017UMN145.1 649 ........................................................................................................................

2017UMN296.1 721 ........................................................................................................................

2017UMN834.1 680 ........................................................................................................................

2017UMN991.6 634 ........................................................................................................................

2017UMN1049.1 675 ........................................................................................................................

2017UMN1161.8 721 ........................................................................................................................

2015UMN1635.9 720 ........................................................................................................................

2017KSU1528.8 720 ........................................................................................................................

2015UMN2473.3 674 ........................................................................................................................

2014KSU27.8 720 .........................................G..............................................................................

2015UMN2626.7 626 ........................................................................................................................

2015KSU2327.2 721 ........................................................................................................................

2016KSU47.5 721 ........................................................................................................................

2015UMN2795.3 721 ........................................................................................................................

2015UMN2869.3 649 ........................................................................................................................

2015UMN3222.1 670 ........................................................................................................................

2014UMN3951.1 720 ........................................................................................................................

2015UMN43.3 645 ........................................................................................................................

2015UMN193.3 720 ........................................................................................................................

2015UMN367.3 721 ........................................................................................................................

2015UMN370.6 720 .........................................G..............................................................................

2015UMN1919.5 674 ........................................................................................................................

2014UMN3749.9 669 ........................................................................................................................

2015UMN64.3 721 .........................................G..............................................................................

2015UMN277.7 649 ........................................................................................................................

2015UMN1735.7 721 ...............................................................G........................................................

2014UMN3631.1 641 ........................................................................................................................

2014UMN3930.3 721 ........................................................................................................................

2015UMN4145.9 659 ........................................................................................................................

2015UMN4053.5 721 ........................................................................................................................

05ZYH33 841 ATAGCTTTAACAAGGTCGTTAAGCTCAAGGATAGTAGCTTCTTTAATTTCAGCAATAATGTTTTCAATGTTCAATGCCATTGTGATTTTCCTCCAATTTAGGTTTTTAAATAGTTTGTAG

2016UMN1917.1 841 ........................................................................................................................

2014UMN2148.7,

2015UMN3436.5,

2014UMN1785.4,

2015UMN56.5, and

2016UMN1470.8 841 ........................................................................................................................

2016UMN1440.1 840 ........................................................................................................................

2016UMN917.8 and

2014UMN3704.91B 789 ........................................................................................................................

2014UMN3624.74 and

2014UMN3714.6 841 ........................................................................................................................

2016UMN102.5 793 ........................................................................................................................

2016UMN146.4 818 ........................................................................................................................

2016UMN209.8 765 ........................................................................................................................

2014UMN3786.4 830 ........................................................................................................................

2016UMN668.5 841 ........................................................................................................................

2014UMN3368.9 and

2016UMN924.3 794 ........................................................................................................................

2016UMN1367.1 and

2017UMN144.4 841 ........................................................................................................................

2015UMN3869.3 808 ........................................................................................................................

2015UMN592.5,

2014UMN3720.5,

2015UMN865.5, and

2016UMN2465.63B 841 ........................................................................................................................

2016UMN2500.63 841 ........................................................................................................................

2016UMN2500.62B 759 ........................................................................................................................

2014UMN3928.6 784 ........................................................................................................................

2015UMN27.5 780 ........................................................................................................................

2015UMN122.1 817 ........................................................................................................................

2017UMN355.91 769 ........................................................................................................................

2016UMN1524.93 841 ........................................................................................................................

2017UMN236.4 841 ........................................................................................................................

2014UMN3624.71,

2017UMN631.2,

2016UMN2813.1,

2016UMN3027.6,

2017UMN55.9,

2017UMN164.7,

2017UMN1574.421, and

2014KSU2091.1 841 ........................................................................................................................

2015UMN1502.6,

2015UMN1502.7,

2016UMN2677.3, and

2017UMN539.9 841 ........................................................................................................................

2016UMN2940.9 766 ........................................................................................................................

2016UMN3248.4 790 ........................................................................................................................

2016UMN3250.3 789 ........................................................................................................................

2017UMN145.1 769 ........................................................................................................................

2017UMN296.1 841 ........................................................................................................................

2017UMN834.1 800 ........................................................................................................................

2017UMN991.6 754 ........................................................................................................................

2017UMN1049.1 795 ........................................................................................................................

2017UMN1161.8 841 ........................................................................................................................

2015UMN1635.9 840 ........................................................................................................................

2017KSU1528.8 840 ........................................................................................................................

2015UMN2473.3 794 ........................................................................................................................

2014KSU27.8 840 ........................................................................................................................

2015UMN2626.7 746 ........................................................................................................................

2015KSU2327.2 841 ........................................................................................................................

2016KSU47.5 841 ........................................................................................................................

2015UMN2795.3 841 ........................................................................................................................

2015UMN2869.3 769 ........................................................................................................................

2015UMN3222.1 790 ........................................................................................................................

2014UMN3951.1 840 ........................................................................................................................

2015UMN43.3 765 ........................................................................................................................

2015UMN193.3 840 ........................................................................................................................

2015UMN367.3 841 ........................................................................................................................

2015UMN370.6 840 ........................................................................................................................

2015UMN1919.5 794 ........................................................................................................................

2014UMN3749.9 789 ........................................................................................................................

2015UMN64.3 841 ........................................................................................................................

2015UMN277.7 769 ........................................................................................................................

2015UMN1735.7 841 ........T...............................................................................................................

2014UMN3631.1 761 ........................................................................................................................

2014UMN3930.3 841 ........................................................................................................................

2015UMN4145.9 779 ........................................................................................................................

2015UMN4053.5 841 ........................................................................................................................

05ZYH33 961 CACTAAGCAGCTACGC

2016UMN1917.1 961 ................

2014UMN2148.7,

2015UMN3436.5,

2014UMN1785.4,

2015UMN56.5, and

2016UMN1470.8 961 ..............--

2016UMN1440.1 960 ..............--

2016UMN917.8 and

2014UMN3704.91B 909 ................

2014UMN3624.74 and

2014UMN3714.6 961 ................

2016UMN102.5 913 ................

2016UMN146.4 938 ..............--

2016UMN209.8 885 ..............--

2014UMN3786.4 950 ................

2016UMN668.5 961 ................

2014UMN3368.9 and

2016UMN924.3 914 ................

2016UMN1367.1 and

2017UMN144.4 961 ................

2015UMN3869.3 928 ................

2015UMN592.5,

2014UMN3720.5,

2015UMN865.5, and

2016UMN2465.63B 961 ..............--

2016UMN2500.63 961 ................

2016UMN2500.62B 879 ..............--

2014UMN3928.6 904 ................

2015UMN27.5 900 ................

2015UMN122.1 937 ................

2017UMN355.91 889 ..............--

2016UMN1524.93 961 ..............--

2017UMN236.4 961 ..............--

2014UMN3624.71,

2017UMN631.2,

2016UMN2813.1,

2016UMN3027.6,

2017UMN55.9,

2017UMN164.7,

2017UMN1574.421, and

2014KSU2091.1 961 ................

2015UMN1502.6,

2015UMN1502.7,

2016UMN2677.3, and

2017UMN539.9 961 ..............--

2016UMN2940.9 886 ................

2016UMN3248.4 910 ................

2016UMN3250.3 909 ................

2017UMN145.1 889 ..............--

2017UMN296.1 961 ..............--

2017UMN834.1 920 ..............--

2017UMN991.6 874 ................

2017UMN1049.1 915 ................

2017UMN1161.8 961 ..............--

2015UMN1635.9 960 ................

2017KSU1528.8 960 ................

2015UMN2473.3 914 ................

2014KSU27.8 960 ................

2015UMN2626.7 866 ................

2015KSU2327.2 961 ................

2016KSU47.5 961 ..............--

2015UMN2795.3 961 ..............--

2015UMN2869.3 889 ................

2015UMN3222.1 910 ................

2014UMN3951.1 960 ..............--

2015UMN43.3 885 ................

2015UMN193.3 960 ..............--

2015UMN367.3 961 ..............--

2015UMN370.6 960 ................

2015UMN1919.5 914 ................

2014UMN3749.9 909 ................

2015UMN64.3 961 ................

2015UMN277.7 889 ................

2015UMN1735.7 961 ................

2014UMN3631.1 881 ..............--

2014UMN3930.3 961 ................

2015UMN4145.9 899 ................

2015UMN4053.5 961 ................
